# Whole Genome Sequencing and Characteristics of *mcr*-1–Harboring Plasmids of Porcine *Escherichia coli* Isolates Belonging to the High-Risk Clone O25b:H4-ST131 Clade B

**DOI:** 10.3389/fmicb.2020.00387

**Published:** 2020-03-24

**Authors:** Saskia-Camille Flament-Simon, María de Toro, Azucena Mora, Vanesa García, Isidro García-Meniño, Dafne Díaz-Jiménez, Alexandra Herrera, Jorge Blanco

**Affiliations:** ^1^Laboratorio de Referencia de E. coli (LREC), Departamento de Microbiología y Parasitología, Facultad de Veterinaria, Universidad de Santiago de Compostela, Lugo, Spain; ^2^Instituto de Investigación Sanitaria de Santiago de Compostela (IDIS), Santiago, Spain; ^3^Plataforma de Genómica y Bioinformática, Centro de Investigación Biomédica de La Rioja (CBIR), Logroño, Spain

**Keywords:** *Escherichia coli*, ST131, swine, colistin, *mcr-1.1*, core genome, plasmidome

## Abstract

Porcine *Escherichia coli* ST131 isolates are scarcely documented. Here, whole genome sequencing and core genome (CG) and plasmidome analysis of seven isolates collected from diarrheic piglets and four from pork meat were performed. All of the 11 ST131 isolates belonged to serotype O25b:H4 and clade B and showed *fimH*22 allele or mutational derivatives. The 11 porcine isolates possessed virulence traits that classified the isolates as avian pathogenic, uropathogenic, and extraintestinal pathogenic *E. coli*–like (APEC-, UPEC-, and ExPEC-like) and constituted virotype D. The CG was performed for all porcine isolates in addition to 73 ST131 reference isolates from different origins. Within clade B, the CG showed nine subclusters, allowing us to describe five new subclades (B6, B6-like, B7, B8, and B9). There was an association between subclade B6, PST43, virotype D2, and food origin, whereas subclade B7 included PST9 isolates with virotype D5 from diarrheic piglets (*p* = 0.007). The distance between human and porcine isolates from subclades B6 and B7 had an average of 20 and 15 SNP/Mb, respectively. [F2:A-:B1]-IncF, ColE1-like, and IncX plasmids were the most prevalent. Besides, IncF plasmids harbored a ColV region frequent among APEC isolates. Antimicrobial resistance genes conferring resistance to penicillin, tetracycline, quinolones, and colistin were the most common. The *mcr*-1.1 gene was detected in 5 of 11 porcine isolates, integrated into the chromosome of one isolate and into plasmids in the remainder isolates (two MOB_*H*__11_/IncHI2-ST4, one MOB_*P*__3_/IncX4, and one MOB_*F*__12_/IncF [F2:A-:B1] supposedly cointegrated with an IncHI2). The surrounding environments of the *mcr*-1 cassette showed variability. However, there were conserved structures within the same plasmid family. In conclusion, CG analysis defined five new subclades. The ST131 porcine isolates belonged to new subclades B6 and B7. Moreover, porcine and clinical human isolates were strongly related. The 11 porcine ST131 isolates harbored a wide variety of plasmids, virulence, and resistance genes. Furthermore, epidemic plasmids IncX4 and IncHI2 are responsible for the acquisition of *mcr*-1.1 gene. We hypothesize that the APEC-IncF plasmid acquired the *mcr*-1.1 gene via cointegrating an IncHI2 plasmid, which is worrying due to combination of virulence and resistance attributes in a single mobile genetic element.

## Introduction

*Escherichia coli* exists as part of the commensal microbiota in the mammalian digestive tract, as well as a zoonotic pathogen responsible for intestinal and extraintestinal infections in both humans and animals (Poirel et al., 2018). Treatment of multidrug-resistant (MDR) *E. coli* infections has become a serious clinical issue, especially with the emergence of high-risk clones, such as clone ST131 (Nicolas-Chanoine et al., 2008, 2014).

ST131 clone is one of the most prevalent extraintestinal pathogenic *E. coli* clones (ExPEC). Although it remains unclear why this clone is prevailing over the others, antimicrobial resistance and virulence factors are suspected contributors (Dobrindt, 2005; Stoesser et al., 2016b). Even though ST131 clone is prevalent in human samples, it remains less frequent from animals and especially rare from porcine source (Reid et al., 2019). However, it has already been identified in many animal species, including in Antarctic pinnipeds (Mora et al., 2018). The majority of animal isolates belong to clade B (associated with *fimH22*), whereas in those that cause extraintestinal infections in humans predominate clade C (associated with *fimH30*) and especially those belonging to subclades C1-nM27, C1-M27, and C2 (H30Rx). This could be the reason of the underrepresentation of ST131 clade B isolates in the literature and sequence databases (Reid et al., 2019).

Worryingly, resistance to last-resort antibiotics such as carbapenems and polymyxins has already been reported in the pandemic *E. coli* clone ST131 (Trobos et al., 2009; Schink et al., 2013; Hasman et al., 2015; Ewers et al., 2016; Kuo et al., 2016; Sonnevend et al., 2016; de Toro et al., 2017; Ortiz de la Tabla et al., 2017; García-Meniño et al., 2018; Liu H. et al., 2018; Ellaby et al., 2019; Hojabri et al., 2019; Reid et al., 2019). Colistin (polymyxin E) is associated with nephrotoxicity and neurotoxicity. However, it has been widely used in veterinary medicine. Livestock, and particularly porcine farming, has been singled out as reservoir for colistin resistance (García-Meniño et al., 2019) and foodborne pathogens (FBPs) that could have devastating health and economic consequences (Sekse et al., 2017).

Intrinsic resistance to colistin has been related with two chromosomally encoded systems, the PhoPQ component and the *pmr*CAB operon (Olaitan et al., 2014; Poirel et al., 2018). However, the newly discovered plasmid-borne mobile colistin resistance (*mcr*) gene is responsible for a transferable mechanism of resistance (Liu Y.-Y. et al., 2016). Since the description of the *mcr*-1 gene by Liu Y.-Y. et al. (2016), several amino acid variants have been described, encoded in *mcr*-2 to *mcr*-9 genes (AbuOun et al., 2017; Borowiak et al., 2017; Carattoli et al., 2017; Yin et al., 2017; Wang et al., 2018; Yang et al., 2018; Carroll et al., 2019). Besides, several subvariants for some of them have been reported, that is, 13 nucleotide and protein variants in the *mcr*-1 family (designated *mcr*-1.1 to *mcr*-1.13) (Partridge et al., 2018).

Colistin plasmid-borne resistance has widely spread geographically (Kempf et al., 2016). It has been described in numerous genera of *Enterobacteriaceae* including *Escherichia*, *Moraxella*, *Klebsiella*, *Salmonella*, *Enterobacter*, *Cronobacter*, *Shigella*, *Kluyvera*, *Citrobacter*, and *Raoultella* (Campos et al., 2016; Liu B.-T. et al., 2016; Liu Y.-Y. et al., 2016; Olaitan et al., 2016; Pham Thanh et al., 2016; Stoesser et al., 2016a; Zeng et al., 2016; Zhao and Zong, 2016; AbuOun et al., 2017; Li et al., 2017c; Luo et al., 2017). Although *mcr* genes have been found in a large diversity of clones, Matamoros et al. (2017) and García-Meniño et al. (2019) established the ST10 and ST155 *E. coli* clones as potential reservoirs of the *mcr*-1 gene.

The *mcr* gene has been detected in many plasmid types, including IncI2, IncHI2, IncP, IncX4, IncY, IncF, and ColE10-like ones (Madec and Haenni, 2018) from different origins (Sun et al., 2018). Nevertheless, Matamoros et al. (2017) and García-Meniño et al. (2019) found that the majority of the *mcr*-carrier plasmids belonged mainly to four plasmid incompatibility groups: IncX4, IncI2, IncHI2, and ColE10-like. In contrast, it remains rare to find colistin resistance genes chromosomally encoded, in accordance to Li et al. (2018) studies, where the prevalence of chromosomal *mcr* carrier isolates was estimated in 4% of the analyzed isolates.

Broadly, the *mcr-1* cassette is described as an approximately 2,600 base pair (bp) fragment containing the *mcr-*1 gene followed by a phosphoesterase (Poirel et al., 2016). It has been proposed that the IS*Apl1* insertion sequence (IS) mediates the transmission of *mcr*-1 by forming circular intermediates, which can translocate (Tegetmeyer et al., 2008; Chandler and Siguier, 2013; Snesrud et al., 2016; Zurfluh et al., 2016a, b; Li et al., 2017a).

Genomic tools allow a compressive characterization of FBPs and the identification of clonal groups of bacteria that represent public health hazards (Kovac et al., 2017). However, epidemiological surveillance of epidemic plasmids related to antimicrobial resistances or/and virulence genes spreading in the bacterial population is still complicated because of the intrinsic plasticity of plasmids (Orlek et al., 2017).

In this study, we have performed the whole genome sequencing (WGS) analysis of 11 ST131 *E. coli* isolates from porcine samples. Our objectives were (I) perform a core genome (CG) analysis to establish the phylogenetic relationship of our isolates within clade B of ST131 *E. coli* isolates, (II) to determine and describe the genetic location of the resistance and virulence genes, (III) to investigate the role of mobile genetic elements (MGEs) in the dissemination of those genes, and (IV) to explore the genetic environment of the *mcr*-1 gene. As far as we know, this study would be the first one in which the whole genome of an extended collection of ST131 clade B isolates from porcine origin, collected from diseased animals and food, has been performed.

## Materials and Methods

### Epidemiological Background of the *E. coli* Collection

In the present study, we performed WGS analysis of a collection of 11 resistant *E. coli* O25b:H4-B2-ST131 clade B isolates, seven from piglets with diarrhea and four from pork meat. The isolates belong to extensive epidemiological studies accomplished in Spain. The seven isolates from diarrheic piglets were isolated during period 2006–2016 (García-Meniño et al., 2018) and the four from pork meat during the years 2011 and 2012 (Herrera Estévez, 2015).

### Antimicrobial Susceptibility Testing

Antimicrobial susceptibility was determined by minimal inhibitory concentrations by using the MicroScan WalkAway^®^ -automated system (BECKMAN COULTER, Inc., Brea, CA, United States) according to the manufacturer’s instructions. The antibiotics tested were ticarcillin, aztreonam, ceftazidime, cefepime, ampicillin–sulbactam, piperacillin–tazobactam, imipenem, meropenem, amikacin, gentamicin, tobramycin, levofloxacin, ciprofloxacin, trimethoprim–sulfamethoxazole, fosfomycin, colistin, minocycline, and tigecycline. Additionally, resistance to ampicillin, cefotaxime, chloramphenicol, and nalidixic acid was determined by disk diffusion assays (Becton Dickinson, Sparks, MD, United States). All results were interpreted according to the CLSI guidelines (Wayne, 2017). Multidrug resistance status was attributed to those isolates resistant to at least one agent of three or more different antimicrobial categories, including resistance to β-lactamase inhibitors (Magiorakos et al., 2012).

### Serotyping, Phylogenetic Grouping, Multilocus Sequence Typing, CH Typing, and Virulence Genotyping

ST131 isolates were characterized with regard to O:H serotypes, phylogenetic groups, clonotypes (*fumC* and *fimH* genes), sequence type by multilocus sequence typing (MLST) (according to the Achtman scheme and by the Pasteur Institute scheme), and 34 extraintestinal virulence-associated genes encoding adhesins (*fimH*, *fimAv*_MT__78_, *papAH*, *papC*, *papEF*, *papGII*, *papGIII*, *sfa/focDE*, *afa/draBC*, *yfcV*), toxins (*sat*, *cnf1*, *hlyA*, *hlyF*, *cdtB*, *tsh*, *vat*), siderophores-iron uptake (*iucD*, *iutA*, *iroN*, *fyuA*, *chuA*), capsule (*kpsM II*, *kpsM II-K2*, *kpsM II-K5*, *neuC-K1*, *kpsM III*), and miscellaneous [*cvaC*, *iss*, *traT*, *ibeA*, *malX* (PAI), *usp*, *ompT*] as described previously (Clermont et al., 2013; Dahbi et al., 2014; Mamani et al., 2019). Based on the definitions given by Johnson et al. (2008, 2015) and Spurbeck et al. (2012), the isolates that genetically satisfied the following criteria (I) positive for ≥2 of 5 markers, including *papAH* and/or *papC*, *sfa/focDE*, *afa/draBC*, *kpsM II*, and *iutA*; (II) positive for three or more of four markers, including *chuA*, *fyuA*, *vat*, and *yfcV*; and (III) positive for ≥4 of 5 markers, including *hlyF*, *iutA*, *iroN*, *iss*, and *ompT*, were presumptively designated as ExPEC, uropathogenic *E. coli* (UPEC), and avian pathogenic *E. coli* (APEC)–like isolates, respectively, taking into account that their site of isolation (meat and feces) and animal species differ from the original definitions. The virotypes (A–F) of the ST131 isolates were established according to the scheme described by Dahbi et al. (2014). Primers used in this study for polymerase chain reaction (PCR) amplification of virulence genes are indicated in Supplementary Table S1.

### WGS, Assembly, and Primary Analysis

Total DNA was extracted with the QIAmp DNA Mini Kit (Qiagen GmbH, Qiagen Strasse 1, Hilden, Germany). Libraries were prepared by using the TruSeq DNA PCR-Free protocol (Illumina, San Diego, California, United States) at the Genomics and Bioinformatics Core Facility (Centre for Biomedical Research of La Rioja). Paired-end 100-bp reads on fragments of 550-bp insert size were sequenced in an Illumina HiSeq 1500. Genomes were reconstructed by using PLACNETw (Vielva et al., 2017). Identification of Open Reading Frames (ORFs) and genome annotation of the assembled genetic elements was performed by using Prokka (Seemann, 2014). Genomes were *in silico* typed by the following databases: SerotypeFinder (Joensen et al., 2015), MLSTtyper (Larsen et al., 2012), and CHtyper (Camacho et al., 2009). Three different databases were used for the identification of antibiotic resistance genes: ResFinder (Zankari et al., 2012), CARD Resistance Gene Identifier (McArthur et al., 2013), and ARG-ANNOT (Antibiotic Resistance Gene-ANNOTation) (Gupta et al., 2014). Besides, the PointFinder database (Zankari et al., 2017) was used to determine point mutations. The VirulenceFinder (Ren et al., 2017) and the VFDB (Chen et al., 2005) databases were used to explore virulence factors.

The genetic environment of the *mcr* detected genes was manually revisited and compared to previously reported ones with CLC Sequence Viewer (version 8.9; Qiagen) and EasyFig tools (Sullivan et al., 2011).

### CG and Phylogenetic Analysis

For phylogenetic analysis, we used the 11 genomes of the porcine *E. coli* sequenced in this study plus 73 full-genomes references from the ST131 clone retrieved from the Enterobase^1^ and the NCBI Bioproject Database.^2^ The CG was defined as the collection of genes present at least once in all the ST131 genomes analyzed, with more than 90% similarity and 90% coverage, as defined by Lanza et al. (2014). All 84 genomes used in this study and their accession number are available on Supplementary Table S2. The analysis included genomes representative of all clades and subclades described by Ben Zakour et al. (2016) and Matamoros et al. (2017).

### Plasmid Analysis

Plasmid reconstruction from WGS data was performed by PLACNETw method (Vielva et al., 2017). In PLACNETw representation, most plasmids can be recognized by their replication initiation proteins (RIPs) and/or RELaxase proteins (REL), both of them considered as plasmid markers. Incompatibility groups (Inc) and pMLST subtypes were *in silico* determined with PlasmidFinder and pMLST (Carattoli et al., 2014), respectively, via the CGE online services.^3^ Besides, reconstructed plasmids were subtyped according to the exact relaxase family subtype by phylogenetic comparison with previously defined relaxases subfamilies (Alvarado et al., 2012). Reconstructed plasmids and references belonging to the same Inc groups were compared by using BRIG (Alikhan et al., 2011) and EasyFig tools (Sullivan et al., 2011). All reference plasmids used in this study were recovered from NCBI database^4^ (Supplementary Table S3).

### Statistics

Normality test was performed (Shapiro–Wilk test). A non-parametric Mann–Whitney–Wilcoxon test on paired data was conducted. A significance level of 0.05 was used for all tests. All statistical analyses were carried out with XLSTAT software.^5^

## Results

### Pasteur Sequence Types and *fimH* Alleles

All of the 11 ST131 isolates belonged to serotype O25b:H4 and the phylogenetic group B2. The seven isolates from piglets with diarrhea showed the Pasteur sequence type (PST) 9, whereas the four isolates from pork meat showed PST43 (Table 1).

**TABLE 1:** Features, molecular typing, virulence, and resistance profiles of ST131 *Escherichia coli* isolates from porcine origin.

Isolate	Origin (year)	Serotype	Phylogroup^a^	Clonotype^b^	MLST^c^	Virulence profile^d,e^	Virotype^f^	Resistance phenotype^g^	MDR + I^h^
LREC_153 (FV11838)	Diarrhea (2008)	O25:H4	B2	CH40-332	ST131/PST9	*iro*N, *iss*, *hly*F, *omp*T, *pap*C, *pap*AH, *pap*EF (GIII), *kpsM*-II (K5), *iuc*D, *iut*A, *chu*A, *fyu*A, *yfc*V, *cva*C, *tra*T, *malX*, *ibeA*, *usp*, *cnf1*, *hly*A	D5	AMC, AMP, AMP/SAM, CZ, GEN, NAL, TI, TOB, TMP/SMX	+
LREC_154 (FV9067)	Diarrhea (2006)	O25:H4	B2	CH40-22	ST131/PST9	*iro*N, *iss*, *hly*F, *omp*T, *pap*C, *pap*AH, *pap*EF (GIII), *kpsM*-II (K5), *iuc*D, *iut*A, *chu*A, *fyu*A, *yfc*V, *cva*C, *tra*T, *malX*, *ibeA*, *usp*	D-nt^*i*^	AMC, AMP, AMP/SAM, CHL, COL, NAL, TI	+
LREC_155 (FV14441)	Diarrhea (2010)	O25:H4	B2	CH40-374	ST131/PST9	*iro*N, *iss*, *hly*F, *omp*T, *pap*C, *pap*AH, *pap*EF (GIII), *kpsM*-II (K5), *iuc*D, *iut*A, *chu*A, *fyu*A, *yfc*V, *cva*C, *tra*T, *malX*, *ibeA*, *usp*, *cnf1*, *hly*A	D5	AMC, AMP, AMP/SAM, TI	−
LREC_157 (FV14983)	Diarrhea (2010)	O25:H4	B2	CH40-161	ST131/PST9	*iro*N, *iss*, *hly*F, *omp*T, *pap*C, *pap*AH, *pap*EF (GIII), *kpsM*-II (K5), *iuc*D, *iut*A, *chu*A, *fyu*A, *yfc*V, *cva*C, *tra*T, *malX*, *ibeA*, *usp*, *cnf1*, *hly*A	D5	AMC, AMP, AMP/SAM, NAL, TI	+
LREC_158 (FV15156)	Diarrhea (2010)	O25:H4	B2	CH40-326	ST131/PST9	*iro*N, *iss*, *hly*F, *omp*T, *pap*C, *pap*AH, *pap*EF (GIII), *kpsM*-II (K5), *iuc*D, *iut*A, *chu*A, *fyu*A, *yfc*V, *cva*C, *tra*T, *malX*, *ibeA*, *usp*, *cnf1*, *hly*A	D5	AMC, AMP, AMP/SAM, NAL, TI	+
LREC_159 (FV12310)	Diarrhea (2009)	O25:H4	B2	CH40-338	ST131/PST9	*iro*N, *iss*, *hly*F, *omp*T, *pap*C, *pap*AH, *pap*EF (GIII), *kpsM*-II (K5), *iuc*D, *iut*A, *chu*A, *fyu*A, *yfc*V, *cva*C, *tra*T, *malX*, *ibeA*, *usp*, *cnf1 hly*A	D5	AMC, AMP, AMP/SAM, COL, GEN, MI, NAL, TI, TOB, TMP/SMX	+
LREC_160 (C153-3A)	Meat (2012)	O25:H4	B2	CH40-298	ST131/PST43	*iro*N, *iss*, *hly*F, *omp*T, *pap*C, *pap*AH, *pap*EF (GIII), *kpsM*-II (K5), *iuc*D, *iut*A, *chu*A, *fyu*A, *yfc*V, *cva*C, *tra*T, *malX*, *ibeA*, *usp*, *cdt*B	D2	AMC, AMP, AMP/SAM, CZ, COL, PI/TZP, TI	+
LREC_161 (C187-6A)	Meat (2012)	O25:H4	B2	CH40-22	ST131/PST43	*iro*N, *iss*, *hly*F, *omp*T, *pap*C, *pap*AH, *pap*EF (GIII), *kpsM*-II (K5), *iuc*D, *iut*A, *chu*A, *fyu*A, *yfc*V, *cva*C, *tra*T, *malX*, *ibeA*, *usp*, *cdt*B	D2	AMC, AMP, AMP/SAM, COL, TI, TMP/SMX	+
LREC_162 (FV14984)	Diarrhea (2010)	O25:H4	B2	CH40-336	ST131/PST9	*iro*N, *iss*, *hly*F, *omp*T, *pap*EF (GIII), *kpsM*-II (K5), *iuc*D, *iut*A, *chu*A, *fyu*A, *yfc*V, *cva*C, *tra*T, *malX*, *ibeA*, *usp*, *cnf1*	D5	AMC, AMP, AMP/SAM, CHL, NAL, TI	+
LREC_168 (C41-4A)	Meat (2011)	O25:H4	B2	CH40-298	ST131/PST43	*iro*N, *iss*, *hly*F, *omp*T, *pap*C, *pap*AH, *pap*EF (GIII), *kpsM*-II (K5), *iuc*D, *iut*A, *chu*A, *fyu*A, *yfc*V, *cva*C, *tra*T, *malX*, *ibeA*, *usp*, *cdt*B, *tsh*	D2	AMC, AMP, AMP/SAM, NAL, TI	+
LREC_176 (C84-4A)	Meat (2011)	O25:H4	B2	CH40-22	ST131/PST43	*iro*N, *iss*, *hly*F, *omp*T, *pap*C, *pap*AH, *pap*EF (GIII), *kpsM*-II (K5), *iuc*D, *iut*A, *chu*A, *fyu*A, *yfc*V, *cva*C, *tra*T, *malX*, *ibeA*, *usp*, *cdt*B	D2	AMP/SAM, COL, TI, TMP/SMX	+

*(a) Phylogroups by Clermont et al. (2013); (b) CH, clonotype (*fumC-fimH* alleles); (c) ST, sequence type according to Achtman scheme; PST, sequence type according to the Pasteur scheme; (d) Extraintestinal virulence genes found by PCR; (e) All isolates exhibited the ExPEC, UPEC and APEC status; (f) The virotype was determined by PCR, based on the presence or absence of 13 virulence genes (Dahbi et al., 2014); (g) AMP, ampicillin; AMC, amoxicillin–clavulanic acid; AMP/SAM, ampicillin–sulbactam; CZ, ceftazidime; CHL, chloramphenicol; COL, colistin; GEN, gentamicin, MI, minocycline; NAL, nalidixic acid; TI, ticarcillin; TMP/SMX, trimethoprim–sulfamethoxazole; TOB, tobramycin; PI/TZP, piperacillin–tazobactam; (h) multidrug drug resistance (MDR); being resistant to at least one agent of three or more different antimicrobial categories including resistance to β-lactamase inhibitors (I); (i) nt, non-typable.*

Also all of the 11 ST131 isolates belonged to clade B and showed the *fimH*22 allele or mutational derivatives of the *fimH22* allele (*fimH*161, *fimH*298, *fimH*326, *fimH*332, *fimH*336, *fimH*338, and *fimH*374), resulting in clonotypes CH40-22 (3 isolates), CH40-161 (1), CH40-298 (2), CH40-326 (1), CH40-332 (1), CH40-336 (1), CH40-338 (1), and CH40-374 (1).

#### Virotypes

The 11 ST131 isolates harbored between 17 and 20 virulence gene markers screened by PCR. All isolates harbored the following genes: *iro*N, *iss*, *hly*F, *omp*T, *pap*EF, *papG* III, *kpsM*-II-K5, *iuc*D, *iut*A, *chu*A, *fyu*A, *yfc*V, *cva*C, *tra*T, *malX*, *ibeA*, and *usp.* However, some genes were variables: *pap*C and *pap*AH (10 isolates), *cnf*1 (six isolates), *hly*A (five isolates), and *cdt*B (four isolates). All isolates belonged to virotype D (Table 1). Six of seven isolates recovered from piglets with diarrhea with PST9 showed the virotype D5 (*ibeA*, *papG* III, *cnf1*, *hlyA*, and *kpsM*-II-K5), whereas the four PST43 isolates from meat showed the virotype D2 (*ibeA*, *papG* III, *cdtB*, and *kpsM*-II-K5). All the 11 porcine ST131 isolates exhibited a virulence profile that satisfied the APEC, UPEC, and ExPEC status and because of their origin were classified as APEC, UPEC, and ExPEC like isolates (Table 1 and Supplementary Table S1).

#### Resistance Phenotype

The resistance profile of the 11 ST131 porcine isolates was determined, showing that all but one exhibited an MDR phenotype. Amoxicillin/clavulanic acid resistance was present in 10 isolates, quinolone resistance in seven, colistin resistance in five isolates (with *mcr*-1 gene), cotrimoxazole resistance in four isolates, chloramphenicol resistance in two isolates, aminoglycoside resistance in two isolates, and tetracycline resistance in one isolate (Table 1).

#### Whole Genome Sequencing

The draft genomes of the 11 ST131 porcine isolates yielded 32 to 78 contigs larger than 1 Kb, with assembly sizes ranging from 4.940 to 5.451 Mb (Supplementary Table S4). The WGS data were in complete agreement with our previous characterization performed with conventional methods.

#### C*G* and Phylogenetic Analysis

A total of 84 *E. coli* genomes were taken from different clades and subclades of ST131 in order to calculate the ST131 CG. The main objectives were in regard to insights of ST131 lineage and the relationship between isolates of different origins, with special attention to clade B of ST131 lineage. Because of that, the CG analysis was dominated by clade B (*n* = 49, 58%), and the remainder isolates belonged to clade C (*n* = 26, 31%), clade A (*n* = 8, 10%), and clade I (*n* = 1, 1%). The final collection represented sequences from human (*n* = 51, 61%), porcine (*n* = 19, 23%), avian (*n* = 8, 10%), and wild animals (Antarctic pinnipeds) (*n* = 6, 7%) sources.

**FIGURE 1:**
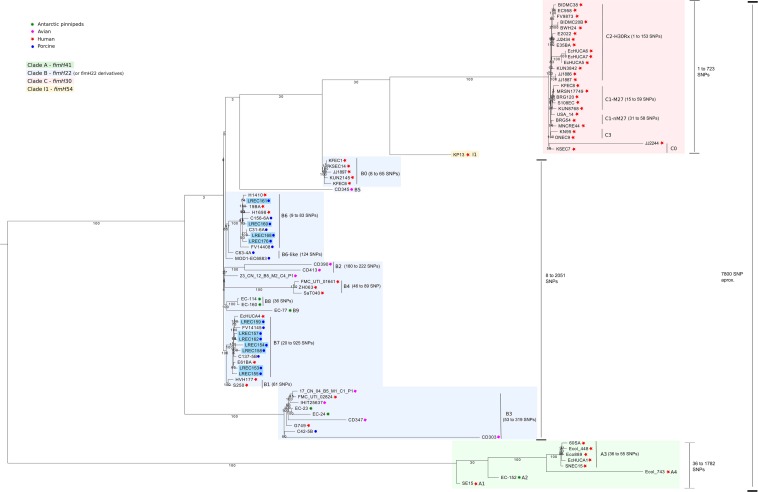
Phylogenetic analysis by core genome of the *Escherichia coli* sequenced in this study (*n* = 11) plus other references (*n* = 73) from ST131 clone.

The CG encompasses 2.98 Mb (length = 2,983,333 ± 7,338 pb), which comprised 3,100 total CDS. The single nucleotide polymorphisms (SNPs) analysis of the CG performed within ST131 lineage revealed a well-defined clade structure, similar to those previously described, including the three defined clades: A, B, and C. In total, 14,838 SNPs were present in the alignment. Furthermore, there was a distance of 7,742 to 8,565 SNPs between isolates from clade A and clade C; 6,508 to 8,675 between isolates from clade A and clade B; and 2,214 to 4,280 between isolates from clade B and clade C. The distance within clades varies greatly: being 36 to 1,782 SNPs in clade A, 8 to 2,051 SNPs in clade B, and 1 to 723 SNPs in clade C (Figure 1 and Supplementary Table S2).

All isolates of porcine origin analyzed in this study were grouped in clade B. As shown in Figure 1, clade B was characterized by the presence of a mixture of human, livestock, and food origin genomes and showed the greatest intragroup diversity. We identified 11 clusters or subclades (B0, B1, B2, B3, B4, B5, B6, B6-like, B7, B8, and B9), including three subclades (B3, B6, and B7) with isolates of both human and animal origin. In this study, we described five new subclades, named B6, B6-like, B7, B8, and B9. There was a distance of 167 to 232 SNPs between isolates from B6 and B6-like and 172 to 304 SNPs between isolates from B6 and B7. Further, B7 showed 119 to 145 SNPs distance with B1. Those subclades appeared as the closer within each other in clade B (Supplementary Table S2). The 11 ST131 porcine isolates from this study were grouped in the new subclades B6 (LREC_160/PST43, LREC_161/PST43, LREC_168/PST43, LREC_176/PST43 from pork meat and virotype D2) and B7 (LREC_153/PST9, LREC_154/PST9, LREC_155/PST9, LREC_157/PST9, LREC_158/PST9, LREC_159/PST9, LREC_162/PST9 from piglets with diarrhea and virotype D5). The new subclade B6-like includes two isolates from porcine origin. Otherwise, new subclades B8 and B9 only include ST131 isolates from Antarctic pinnipeds.

Notably, there were three (H1410, 19BA, H1698) isolates from human origin in subclade B6 and two (EcHUCA4, E61BA) in subclade B7. The distance between human isolates and porcine isolates from subclades B6 and B7 had an average of 60 SNPs and of 46 SNPs (i.e., 20 and 15 SNP/Mb), respectively.

#### Plasmidome Analysis

The 11 ST131 porcine isolates showed high heterogeneity in their plasmid content, harboring between two and seven different plasmids (Table 2 and Supplementary Figure S1). We find members of eight of the 17 main MOB plasmid groups found in γ−proteobacteria, ordered by prevalence: MOB_F__12_ (*n* = 13), MOB_P__51_ (*n* = 8), MOB_P__3_ (*n* = 3), MOB_V__2_ (*n* = 2), MOB_H__11_ (*n* = 2), MOB_P__12_ (*n* = 2), MOB_P__131_ (*n* = 1), and MOB_Q__12_ (*n* = 1). Finally, we described six plasmids (16%) in which any MOB protein was found (non-mobilizable-infective plasmids). Twenty-two plasmids were considered large (>33 kb) and presumptively conjugative, and sixteen were small multicopy plasmids (<7 kb).

**TABLE 2:** Plasmid content of the ST131 *Escherichia coli* isolates from porcine origin and location of resistance and virulence genes.

Isolate	Genetic element^a^	No. of Contigs	Size (bp)	No. of copies^b^	Relaxase protein^c^	Replication protein^c^	Inc group (pMLST)^d^	Virulence genes^e^	Resistence genes^f^, point mutations and efflux/transporter genes
LREC_153	chr_LREC153	40	4,904,938	1	nd^h^	nd	na^g^	*papACDEFHJKX, hlyABD, cnf1, chuA*	PBP,*AmpH, gyrA_S83L, mdfA*
	pLREC153_1	8	330,910	1	MOB_F12_, MOB_H11_	RptA1	IncF [F2:A-:B1][IncHI2-ST4]^i^	*iroBCDEN, iss, mchF, iutA, iucABCD*	
	pLREC153_2	16	102,056	1	MOB_*P131*_	RptZ, RptC	IncL/M [IncQ1]^*i*^		*bla_*TEM–1A*_/bla_TEM–54_/bla_TEM–150_, AAC(3′)-IIa, AAC(3′)-IIc, APH(3′)-Ia, APH(3′’)-Ib, APH(6′)-Id, aadA1, strA, strB, sul1, sul2, sul3, dfrA1, dfrA15, tet(A), tet(C), qacEdelta1*
	pLREC153_3	1	6,851	5	MOB_P51_	nd	ColE1-like		
	pLREC153_4	1*	2,461	4	MOB_V2_	RepL	nd		
LREC_154	chr_LREC154	111	4,946,997	1	nd	nd	na	*papBCDEFHIJKX, iss, iroBCDEN, iucABCD, iutA*	PBP,*AmpH, gyrA_S83L, mdfA, mcr-1.1*
	pLREC154_1	2	124,513	1	MOB_F12_	RptA1	IncF [F2:A-:B1]	*iroBCDEN, iss, mchF, iutA, iucABCD*	
	pLREC154_2	27	56,729	3	MOB_P3_	RptF	IncX4		*bla_TEM–1C_/bla_TEM–40_/bla_TEM–135_, tet(A), tet(C), tet(M), tet(R)*
LREC_155	chr_LREC155	60	4,947,621	1	nd	nd	na	*papACDEFHJKX, hlyABD, cnf1, chuA, mchC, iha, iroBCDEN, iucABCD, iutA*	PBP,*AmpH, mdfA*
	pLREC155_1	25	316,521	1	MOB_F12_	RptA1	IncF [F2:A-:B1][IncHI2-ST-nt]^i^	*iroBCDEN, iss,cma, cba, mchF, iutA, iucABCD*	
	pLREC155_2	23	208,964	1	MOB_P12_	RptZ	IncI1-ST27		*bla_TEM–1C_/bla_TEM–40_/bla_TEM–135,_ tet(A), tet(C), tet(R)*
LREC_157	chr_LREC157	79	4,896,484	1	nd	nd	na	*papACDEFHJKX, hlyABD, cnf1, chuA, iroBCDEN, iucABCD, iutA*	PBP,*AmpH, gyrA_S83L, mdfA*
	pLREC157_1	12	137,775	1	MOB_F12_	RptA1	IncF [F2:A-:B1]	*iroBCDEN, iss, mchF, iutA, iucABCD*	*bla_TEM–1C_/bla_TEM–40_/bla_TEM–135_*
	pLREC157_2	1	46,172	1	MOB_P3_	RptF	IncX1		
	pLREC157_3	1	6,851	3–4	MOB_P51_	nd	ColE1-like		
	pLREC157_4	1*	2,047	5	MOB_V2_	nd (RepA partial hit)	nd		
LREC_158	chr_LREC158	58	4,853,444	1	nd	nd	na	*papACDEFHJKX, hlyABD, cnf1, chuA, iroBCDEN, iucABCD, iutA*	PBP,*AmpH, bla_TEM–1C_/bla_TEM–40_/bla_TEM–135_, gyrA_S83L, mdfA*
	pLREC158_1	5	135,569	1	MOB_F12_	RptA1	IncF [F2:A-:B1]	*iroBCDEN, iss, mchF, iutA, iucABCD*	*tet(A), tet(C), tet(R)*
	pLREC158_2	1*	6,648	4	MOB_P51_	nd	ColE1-like		
	pLREC158_3	1*	1,554	5	nd	RptL2	nd (Cryptic_1)		
LREC_159	chr_LREC159	97	4,869,944	1	nd	nd	na	*papACDEFHJKX, hlyABD, cnf1, chuA, iroBCDEN, iucABCD, iutA*	PBP,*AmpH, gyrA_S83L, mdfA*
	pLREC159_1	10	248,461	1	MOB_H11_	RptC	IncHI2-ST4 [IncQ1]^*i*^		*APH(3′)-Ia, APH(3″)-Ib, APH(6)-Id, strA, strB, sul2, sul3, mcr-1.1*
	pLREC159_2	9	177,070	1	MOB_F12_	RptA1	IncF[F2:A-:B1]	*iroBCDEN, iss, mchF, iutA, iucABCD*	*bla_TEM–1C/_bla_TEM–40/_bla_TEM–135_, aadA1, AAC(3)-IIa, AAC(3)-IIc, sul1, sul2, dfrA1, drfA15,tet(A), tet(C), tet(R), qacEdelta1*
	pLREC159_3	1	68,523	1	MOB_P12_	RptZ	Incl1-ST171		
	pLREC159_4	1	2,014	2	MOB_Q12_	nd	nd		*aadA2, ANT(3″)-Ia, linG, lnu(F)*
	pLREC159_5	1*	1,553	4	nd	RptL2 (HTH36 family)	nd (Cryptic_1)		
	pLREC159_6	1*	1,507	3	nd	nd (RepA partial hit)	nd (Cryptic_2)		
LREC_160	chr_LREC160	107	5,049,024	1	nd	nd	nd	*papCDEFJK, sfaX, mchCF, iha, iss, chuA, iroBCDEN, iucABCD, iutA*	PBP,*AmpH, mdfA*
	pLREC160_1	18	145,187	1	MOB_F12_	RptA1	IncF [F2:A-:B1]	*iroBCDEN, iss, mchF, iutA, iucABCD*	*bla_TEM–1C/_bla_TEM–40/_bla_TEM–135_, tet(A), tet(C), tet(R)*
	pLREC160_2	5	40,221	2	MOB_P3_	RptF	IncX4		*bla_TEM–1C/_bla_TEM–40/_bla_TEM–135_, mcr-1.1*
	pLREC160_3	1	5,630	3	MOB_P51_	nd	ColE1-like		
	pLREC160_4	1	5,006	4–5	MOB_P51_	nd	ColE1-like		
	pLREC160_5	1	4,515	3–4	MOB_P51_	nd	ColE1-like		
	pLREC160_6	1	1,765	3–4	MOB_P51_	nd	ColE1-like		
	pLREC160_7	1*	1,551	4	nd	RptL2	nd (Cryptic_1)		
LREC_161	chr_LREC161	64	5,072,645	1	nd	nd	na	*papCDEFJK, sfaX, iss, chuA, iroBCDEN, iucABCD, iutA*	PBP,*AmpH, mdfA*
	pLREC161_1	8	330,357	1	MOB_F12_	RptA1	IncF [F2:A-:B1][IncHI2-ST-nt]^i^	*iroBCDEN, iss, mchF, iutA, iucABCD*	*bla_TEM–1C/_bla_TEM–40/_bla_TEM–135_, mcr-1.1, tet(A), tet(C), tet(R)*
	pLREC161_2	15	33,473	2	nd	RptF	nd		*bla_TEM–1C_, aadA2, aadA17, ANT(3″)-Ia, sul3, drfA12, linG, lnu(F), mef(B)*
	pLREC161_3	1	6,826	3	MOB_P51_	nd	ColE1-like		
	pLREC161_4	1*	1,553	4	nd	RptL2 (HTH36 family)	nd (Cryptic_1)		
LREC_162	chr_LREC162	46	4,720,511	1	nd	nd	na	*chuA, iroBCDEN, iucABCD, iutA*	PBP,*AmpH, gyrA_S83L, mdfA*
	pLREC162_1+2^J^	29	216,025	1	MOB_F12_ (2) ^J^	RptA1	IncF [F10:A-:B1]	*iroBCDEN, iss, mchF, iutA, iucABCD*	*bla_TEM–1C/_bla_TEM–40/_bla_TEM–135_*
LREC_168	chr_LREC168	113	5,117,208	1	nd	nd	na	*papCDEFJK, sfaX, iss, chuA, iroBCDEN, iucABCD, iutA*	PBP,*AmpH, gyrA_S83L, mdfA*
	pLREC168_1+2^J^	13	144,861	1	MOB_F12_ (2) ^J^	RptA1	IncF [F2:A-:B1]	*iroBCDEN, iss, mchF, iutA, iucABCD*	*bla_TEM–1C/_bla_TEM–40/_bla_TEM–135_, tet(A), tet(C), tet(R)*
LREC_176	chr_LREC176	145	5,039,239	1	nd	nd	na	*papDFJKX, sfaX, iss, chuA, iroBCDEN, iucABCD, iutA*	PBP,*AmpH, mdfA*
	pLREC176_1	15	278,079	1	MOB_H11_	RepFIB_RepA (x2)	IncHI2-ST4		*bla_TEM–1A_/bla_TEM–150_, aadA1, aadA2, APH(6)-Id, APH(3″)-Ib, strA, strB, sul1, sul2, sul3, drfA1, drfA15, mcr-1.1, tet(A), tet(C), tet(R), cmlA1, cmlA6, catA1, qacEdelta1, qacHR*
	pLREC176_2	9	132,494	1	MOB_F12_	RptA1	IncF [F2:A-:B1]	*iroBCDEN, iss, mchF, iutA, iucABCD*	

^*a*^Chr, chromosome; p, plasmid. ^*b*^Approximative number of plasmid copies based on sequencing coverage identified by PLACNET. ^*c*^Relaxase and replication proteins were identified by the use of PLACNET databases. ^*d*^Incompatibility groups were determined according to the PBRT scheme and pMLST subtypes according to the allele scheme in http://pubmlst.org/plasmid/. ^*e*^Virulence genes were detected through VirulenceFinder and VFDB databases (minimum coverage of 90%, minimum identity of 75%); all genomes presented in their chromosome the following genes: *aslA*, *cheBWY*, *chuSTUVWXY*, *csgBDEFG*, *entABCEFS*, *fdeC*, *fepABCDG*, *fes*, *fimABCDEFGHI*, *flgG*, *flhAC*, *fliGIMP*, *fyuA*, *gad*, *gspCDEFGHIJKLM*, *ibeA*, *irp1*, *irp2*, *kpsDM*, *OmpA*, *yagVWXYZ/ecpEDCBA*, *ybtAEPQSTUX*, *ykgK/ecpR*. ^*f*^Antimicrobial resistance genes were detected by using ARG-ANNOT, CARD and ResFinder databases (minimum coverage of 93%, minimum identity of 75%); all genomes presented in their chromosome the following genes (efflux pumps and transport modulators): *acrAEFS*, *bacRS*, *cpxA*, *CRP*, *emrABEKRY*, *eptA*, *evgAS*, *gadWX*, *H-NS*, *kdpE*, *marA*, *mdtABCEFGHKNOP*, *msbA*, *pmrF*, *tolC*, *ugd*, *yojI.*^*g*^na, not-applicable; ^*h*^nd, not detected. ^*i*^Cointegrated plasmid. ^*j*^Two plasmids that could not be separated (two MOB_*F12*__)_.*Closed plasmids.

In summary, 38 plasmids were described in the 11 genomes analyzed. The plasmids belonged to the following relaxase families (MOB) and incompatibility groups (Inc): MOB_P__51_/ColE1-like (*n* = 8), MOB_F__12_/IncF [F2:A-:B1] (*n* = 8), MOB_F__12_/IncF [F2:A-:B1] plus and presumptively cointegrated IncHI2-ST4/ST-nt (not typable) (*n* = 3), MOB_F__12_/IncF [F10:A-:B1] (*n* = 2), MOB_P__12_/IncI1-ST27/ST171 (*n* = 2), MOB_P__3_/IncX4 (*n* = 2), MOB_V__2_/nd (not detectable) (*n* = 2), MOB_H__11_/IncHI2-ST4 (*n* = 1), MOB_H__11_/IncHI2-ST4 plus and presumptively cointegrated IncQ1 (*n* = 1), MOB_P__131_/IncL/M plus and presumptively cointegrated IncQ1 (*n* = 1), MOB_P__3_/IncX1 (*n* = 1), and MOB_Q__12_/nd (*n* = 1). We also localized six plasmids that could not be affiliated with any categories.

All 13 MOB_F__12_/IncF plasmids were carriers of virulence genes. The genes *iro*BCDEN, *iss*, *mch*F, *iut*A, and *iuc*ABCD were constant (Table 2). All the MOB_F__12_/IncF plasmids found in our study were structurally compared by BRIG software, using the pJIE186_2 (NC_020271) plasmid as reference (Supplementary Figure S2). The plasmids analyzed showed extensive sequence similarity over at least 100 kb, which includes not only backbone genes. The comparison emphasizes that all of them share an 80-kb conserved region comprising the virulence genes described previously as well as *cvaABC*, *sitABC*, *ompT*, and *hlyF*.

The eight MOB_P__51_/ColE1-like plasmids identified in this study exhibited a wide size range (1.7–6.8 kb) and were analyzed using the ColE1 plasmid (J01566.1) as reference (Supplementary Figure S3). The plasmids pLREC153_3, pLREC157_3, pLREC158_2, and pLREC161_3 (all of them >6.8 Kb) showed the highest homology to the reference plasmid. All of them carried the colicin E1 *cea* and immunity (*imm*) genes and the entry exclusion (*exc1*-*exc2*) system genes. On the other hand, plasmids from isolate LREC_160 were different among them and with the others, exhibiting a wide size range (1.8–5.6 Kb), and only pLREC160_4 carried the colicin E1 gene.

Three MOB_P__3_/IncX plasmids were present among our isolates. We found pLREC157_2 that belong to the IncX1 subcluster and had a similar backbone than p2ESCUM (NC_011739.1) but codes for a completely different RIP (Supplementary Figure S4), whereas pLREC154_2 and pLREC160_2 belong to the IncX4 subcluster and were analyzed using pSH696_34 (JX258654.1) as a reference (Supplementary Figure S5). Because pLREC160_2 carried the *mcr.1-1* gene, some previously reported IncX4 reference plasmids harboring the *mcr* gene were added. Compared to pSH696_34, plasmid pLREC160_2 and the other *mcr* carrier plasmids (pESTMCR and pICBEC7Pmcr) lacked two important backbone genes: the conjugative coupling protein gene (*traG*) and the replication protein encoding region (*rep*).

Structural comparison of the two MOB_P__12_/IncI1-ST171 plasmids and the single MOB_P__131_/IncL/M plasmid found within our isolates was also performed using pEK204 (EU935740) and pEC743-OXA48 (CP015071.1) as references, respectively. Both showed a high coverage of homology with the reference (Supplementary Figures S6, S7).

We also described five small (1.5 kb) multicopy plasmids in which any relaxase protein was found, categorized as cryptic. Those cryptic no-MOB plasmids were analyzed by using pEC10D (NC_017650.1) as reference (Supplementary Figure S8). BRIG comparison showed that four plasmids were highly similar to pEC10D and codes for the same replication protein (cryptic 1, from the HTH36 superfamily replication proteins), whereas pLREC159_6 codes for a different replication protein and was called cryptic 2 (Table 2).

Finally, we analyzed the MOB_V__2_ plasmids found in the isolates LREC_153 and LREC_157. A BLAST research was performed to identify a reference plasmid, being pEC0674 (MF684783.1) as the closest match. Two MOB_V__2_-like–related plasmids were also included for structural comparison (Supplementary Figure S9). Although no significant similarity was found between these latter two references and the pEC0674, the pLREC153_4 showed similarity with the replication protein, a hypothetical one and the Rec protein from the reference plasmid, whereas the pLREC157_4 only showed similarity to the latter two proteins.

#### Antimicrobial Resistance Genes

Many antimicrobial resistance genes (ARGs) were found in the 11 ST131 porcine isolates (Table 2), including acquired resistance genes, point mutations, and efflux/transporter genes.

Besides constitutive genes (PBP and AmpH, which were present in all isolates), ARGs conferring resistance to penicillin (variants of *bla*_*TEM*_ gene, accounting: *bla*_*TEM–*__1__*C*_, *n* = 10; *bla*_*TEM–*__40_, *n* = 10; *bla*_*TEM–*__135_, *n* = 10; *bla*_*TEM–*__150_, *n* = 2; *bla*_*TEM–*__1__*A*_, *n* = 2; *bla*_*TEM–*__54_, *n* = 1), tetracycline (variants of *tet* gene, accounting: *tet*A, *n* = 9; *tet*C, *n* = 9; *tet*R, *n* = 8; *tet*M, *n* = 1), quinolones (*gyr*A_S83L, *n* = 7), and colistin (*mcr*-1.1, *n* = 5) were the most common. In addition, we described genes conferring resistance to sulfonamides (variants of *sul* gene, accounting: *sul*2, *n* = 4; *sul*3, *n* = 4; *sul*1, *n* = 3), trimethoprim (variants of *drf* gene, accounting: *dfr*A1, *n* = 3; *dfr*A15, *n* = 3; *dfr*A12, *n* = 1), aminoglycosides (different ACCs, APHs, and ANTs genes, accounting: APH-3″-Ib, *n* = 3; APH-6′-Id, *n* = 3; *aad*A1, *n* = 3; ANT-3″-Ia, *n* = 3; *str*A, *n* = 3; *str*B, *n* = 3; APH-3′-Ia, *n* = 2; *aad*A2, *n* = 2; AAC-3′-IIa, *n* = 2; AAC-3′-IIc, *n* = 2; *aad*A17, *n* = 1), phenicols (variants of *cml*A gene, *cat*A1, and *mdf*A gene, accounting: *mdfA*, *n* = 11; *cml*A1, *n* = 1; *cml*A6, *n* = 1; *cat*A1, *n* = 1), and to ammonium quaternary biocide (*qac*Edelta1, *n* = 3; *qac*HR, *n* = 1) (Table 2).

The phenotypic resistance profile in the studied genomes was due to genes carried by plasmids, except for quinolone resistance, which in our study is mediated by a Ser83Leu point mutation found in the chromosomal *gyr*A gene. In the case of LREC_158 isolate, it exhibited the *bla*_*TEM*_ genes (−1c, −40, and −135) variants set into a chromosomal assembled contig. However, these genes were located into IncL/M, IncX4, IncI1, IncF, and IncHI2 in the other analyzed isolates (Table 2).

As shown in Table 1, all but one isolate exhibited an MDR profile. Large conjugative MDR plasmids were identified in four of the 10 isolates. These were typed as MOB_H__11_/IncHI2-ST4 (pLREC159_1 and pLREC176_1), MOB_F__12_/IncF (pLREC159_2, pLREC161_1), and MOB_P__131_/IncL/M (pLREC153_2) and a non-typable one (pLREC161_2). Most common antibiotic resistance profiles encoded by these plasmids are ampicillin/penicillin, aminoglycosides, tetracyclines, sulfonamides, cotrimoxazole, and polymyxins. Finally, we described some drug efflux genes in pLREC159_4 and pLREC161_2 (Table 2).

Class 1 integrons were present in pLREC153_2 (*IntI-attI* [*-sul2* + *aph-*(*3*″)*-Ib* + *aph-*(*6*)*-Id* + *dfrA1* + *aadA1*]-*qacE1*-*sul1*), pLREC159_2 (*IntI-attI* [*dfrA1* + *aadA1*]-*qacE1-sul1*), and pLREC176_1 (*IntI-attI* [*dfrA1* + *aadA1*]-*qacE1*-*sul1*). No class 2 or 3 integrons were detected in our genomes.

#### Colistin Resistance Vehicles and Genetic Environment

Colistin resistance by *mcr* gene was found in five of the analyzed isolates. Therefore, the *mcr*-carrying plasmids were investigated in greater depth. The *mcr*-1.1 gene was located in two MOB_H__11_/IncHI2-ST4, a MOB_P__3_/IncX4 and a MOB_F__12_/IncF [F2:A-:B1], which was theoretically cointegrated with an IncHI2-ST-nt plasmid. Besides, the LREC_154 isolate showed the *mcr-*1.1 in a chromosomal location (Table 2). In this study, any point mutation in the chromosomally encoded genes *pmr*A or *pmr*B was found.

**FIGURE 2:**
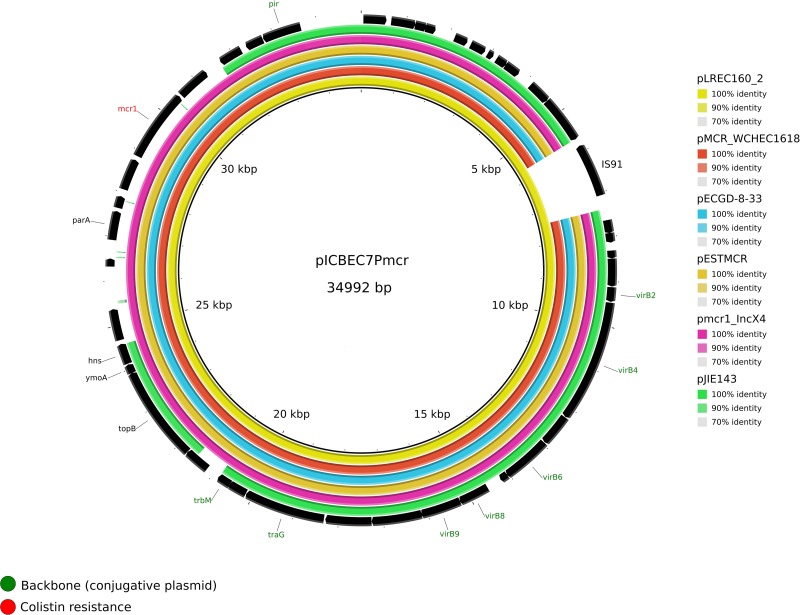
Structural comparison between *mcr*-1 plasmids and IncX4 references. The alignment includes five references and the one IncX4 *mcr*-1–bearing plasmid found in our study. The plasmid pICBEC7*mcr* (IncX4) was used as a reference to match with the other plasmids with [pMCR_WCHEC1618, pECGD-8-33, pESTMCR, and pmcr1_IncX4] and without the *mcr*-1 gene [pIJE143]. The outer circle with black arrows denotes the annotation of reference sequence pICBEC7mcr. The image was generated using BRIG (default parameters with 90/70 as upper/lower threshold).

The complete sequence of pLREC160_2 (MOB_P__3_/IncX4) was 40,221 bp in size and contains 69 predicted ORFs. A structural comparison against other reference IncX4 plasmids is shown in Figure 2, where pICBEC7P*mcr* (34,992 pb, NZ_CP017246.1) was used as the internal reference. The structure of the *mcr-1* carriers IncX4 plasmid remains stable if we exclude one ORF encoding for an IS91 family transposase present in pLREC160_2 and pICBEC7P*mcr*. The BRIG comparison showed that the *mcr-1–*negative plasmid pJIE143 differs from pICBEC7P*mcr* in a region of approximately 6 kb (encoding for *par*A, *mcr*, and *pap*2 genes). All the analyzed plasmids showed a typical IncX4 backbone, including the RIP gene (*pir*), conjugal transfer protein genes (*tra*) plus *taxABC*, and *pilX* operons (Supplementary Figures S10–S13).

**FIGURE 3:**
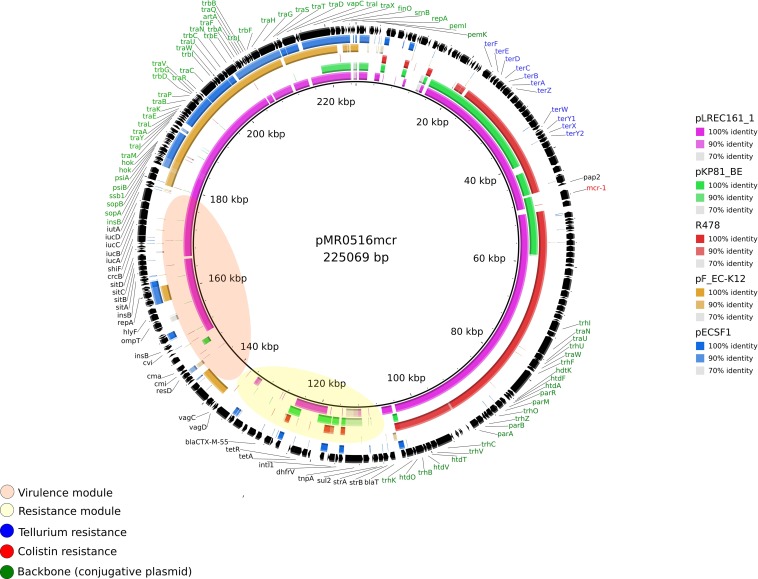
Structural comparison between *mcr-1* plasmids, IncF, and IncHI2 references. The alignment includes four references and the one IncF *mcr*-1–bearing plasmid found in our study. pMR0516mcr (IncF) was used as a reference to match with the other plasmids with [pKP81_BE (IncF)] and without *mcr-1* gene [IncF plasmid from *Escherichia coli* K12, pECSF1 (IncF) and R478 (IncHI2)]. The outer circle with black arrows denotes the annotation of pMR0516mcr. The image was generated using BRIG (default parameters with 90/70 as upper/lower threshold).

Plasmid pLREC161_1 was 330,357 bp in size and contains 616 predicted ORFs. This MOB_F__12_ plasmid belongs to the IncF [F2:A-:B1] incompatibility group and was theoretically cointegrated with an IncHI2 plasmid. A structural comparison with plasmid pMR0516*mcr* [F18:A-:B1] (KX276657.1) as an internal reference (225,069 bp) showed 100% identity in more than 70% of query coverage from BRIG (Figure 3). Genes involved in the conjugative transfer (*tra*, *trb*, and pili operons) in the toxin-antitoxin system (*vagC*) and the inhibition of SOS response (*psi*AB) were present. Most of the differences were observed in the resistance module. It is worthy of note that pLREC161_1 and pMR0516*mcr* plasmids contained APEC virulence genes and that the surrounding region of the *mcr* gene includes heavy metal resistance genes to tellurium (*ter* genes). Moreover, in Supplementary Figure S14, where pKP81_BE [F2:A-:B] (KU994859.1) was used as the reference, we observed 100% of identity in more than 78% of query coverage from BRIG with pLREC161_1. Interestingly, plasmid R478 (IncHI2 reference plasmid, U62007.2) also shared with pKP81_BE some backbone genes and the tellurium resistance module like pMR0516*mcr* (Supplementary Figure S14). A wide part of the backbone in pLREC161_1 (approximately 44 kb) was common with the widely studied IncF plasmid from *E. coli-*K12, used as non-*mcr* plasmid reference (from coord. 55 to 99 kb). The ancestral IncF plasmid of ST131 (pECSF1) was also included in BRIG comparisons (Supplementary Figure S15).

**FIGURE 4:**
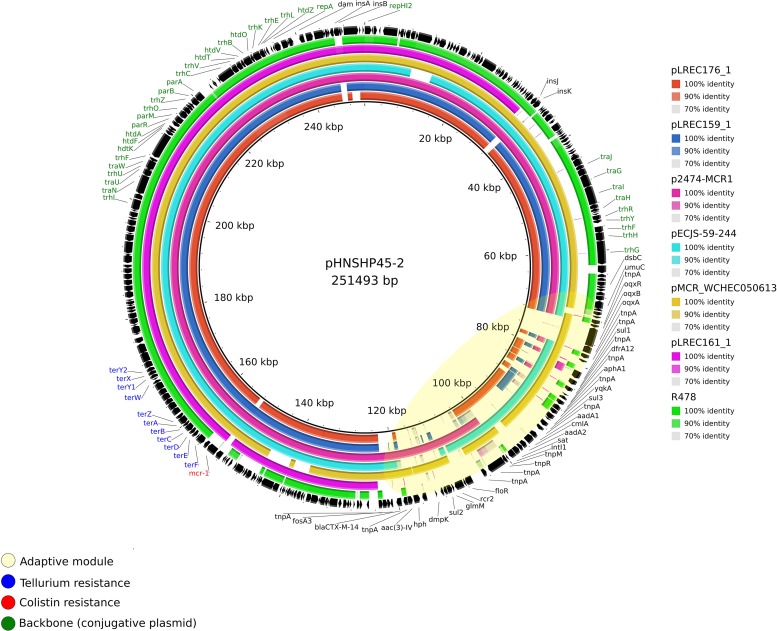
Structural comparison between *mcr-1* plasmids and IncHI2 references. The alignment includes four references and the *mcr*-1–bearing plasmids from our study pLREC176_1 and pLREC159_1, which are IncHI2 and pLREC161_1, which is an IncF plasmid with a presumptively IncHI2 cointegrated. The plasmid pHNSHP45-2 (IncHI2) was used as a reference to match with the other plasmids with [p2474-MCR1 (IncHI2), pECJS-59-244 (IncHI2), and pMCR_WCHEC050613 (IncHI2)] and without the *mcr-1* gene [R478 (IncHI2)]. The outer circle with black arrows denotes the annotation of reference sequence pHNSHP45-2. The image was generated using BRIG (default parameters with 90/70 as upper/lower threshold).

There were two MOB_H__11_/IncHI2-ST4 *mcr-1*–bearing plasmids: (I) pLREC159_1, which was 248,461 bp in size and contains 420 predicted ORFs, and (II) pLREC176_1, which was 278,079 bp in size and contains 523 predicted ORFs. Both showed 100% of identity over more than 75% of query coverage in BRIG comparison with pHNSHP45-2 (KU341381.1) (Figure 4). The backbone was almost identical; nevertheless, the resistance module was distinct between them. The result of this comparative analysis showed that a wide range of resistance genes could be found in the MDR module of IncHI2 plasmids. Moreover, all the IncHI2 plasmids analyzed presented a tellurium resistance module close to the *mcr*-1 cassette. The similarities between IncHI2 plasmids were high, even though their origin and parental isolate characteristics differ.

Afterward, a comparison between all our *mcr*-1–bearing plasmids was performed with pHNSHP45-2 (MOB_H__11_/IncHI2) as a reference, and as expected, almost none homologous region was found between plasmids from different families (Supplementary Figure S16). Nevertheless, pLREC161_1 (MOBF_12_/IncF [F2:A-:B1] plus IncHI2-ST-nt) plasmid shared a part of his backbone with the reference one, including the module encoding for tellurium resistance and the *mcr* gene, suggesting that the *mcr*-1 cassette was introduced into pLREC161_1 through the cointegrated IncHI2 plasmid.

**FIGURE 5:**
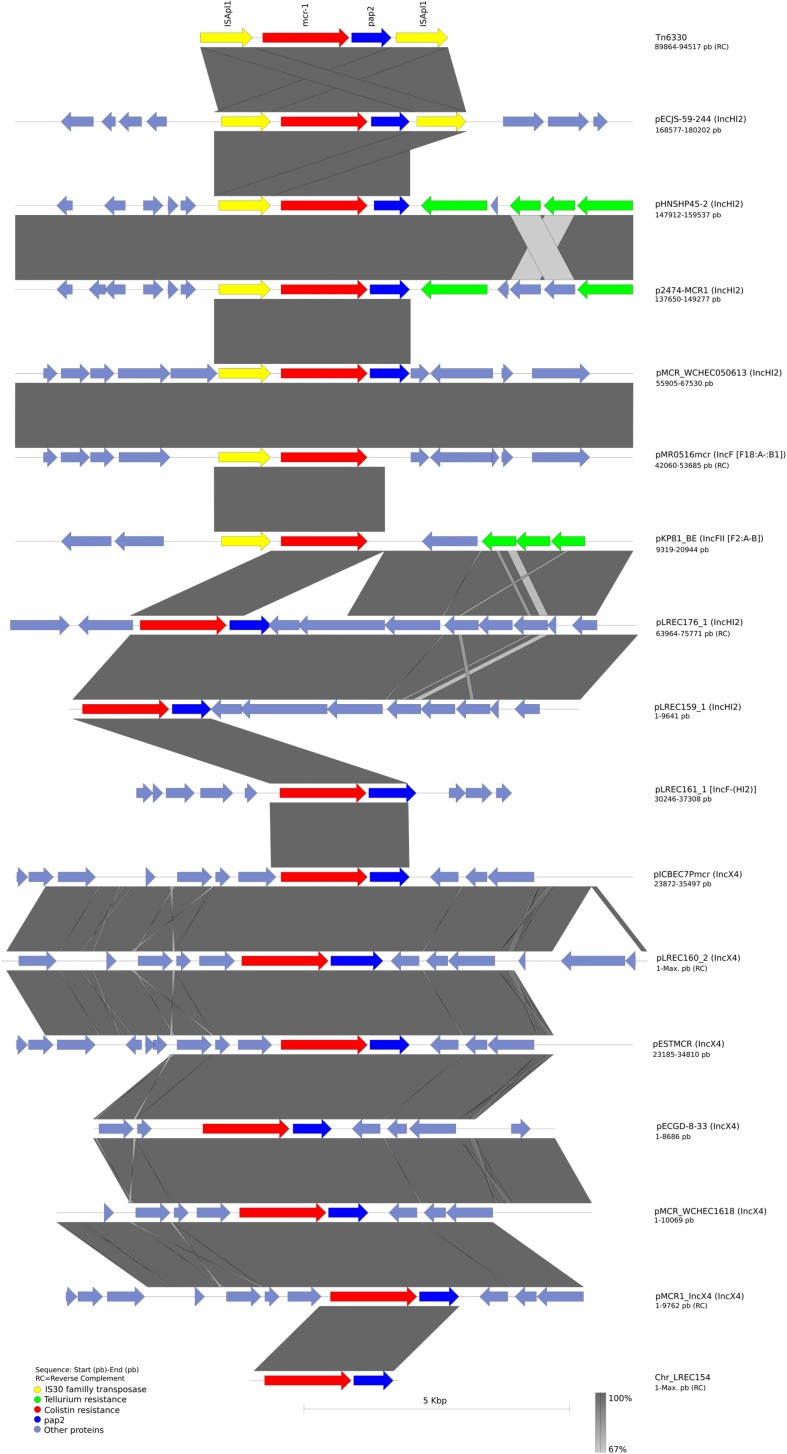
Comparison of the homologous region of the composite transposon Tn6330 (CP029493.1) containing *mcr*-1 gene with our sequences and other frequently used reference sequences analyzed in this article. Open arrows represent coding sequences (green for tellurium resistance, yellow for IS*Apl*1, red for *mcr*-1, and blue for *pap*2) and indicate direction of transcription. The arrow size is proportional to the gene length. The shadow parallelograms denote genetic regions that exhibit sequence homology among different segments. Light shadow denotes regions with a lower level of sequence identity (99%) by BLAST. The image was generated using EasyFig (default parameters).

We had also made a sequence alignment to analyze the surrounding region of our *mcr*-bearing contigs and compare their structure against plasmids references (Figure 5). Regarding plasmid families, all the IncX4 plasmids were identical in terms of the genetic arrangement in the vicinity of the *mcr*-1 gene, whereas the insertion site of *mcr*-1 gene in IncHI2 plasmid was variable. Interestingly, the *mcr* region in plasmids pMCR_WCHEC050613 (IncHI2) and plasmid pMR0516*mcr* (IncF) shared 100% of identity. It is also worth to note that the IncF *mcr* harboring plasmid pKP81_BE carried the tellurite resistance module surrounding the *mcr* gene like the IncHI2 plasmids.

Finally, the genetic arrangement of the *mcr*-1 cassette was investigated (Supplementary Figure S17). All sequences share a 2,542-bp DNA segment, containing *mcr*-1.1 and a putative ORF, encoding a Pap2 superfamily protein. However, some variability in the last 3′ end of the *pap2* gene was observed.

The genome reconstruction of LREC_154 showed that *mcr* gene was inserted into the chromosome. However, the *mcr* harboring contig included only *mcr* and *pap2* genes. It was flanked at both ends by a partial ISA*pl1* sequence of 99 bp upstream and 405 pb downstream the *mcr*-1 cassette. In pLREC159_1, the *mcr* harboring contig was disrupted after a segment of 87 bp of DNA homologous to ISA*pl1* upstream the *mcr* gene. Probably the presence of the IS (repeated element) has interfered with the *de novo* reconstruction of the sequenced genome by the assembly of Illumina short reads. In addition, we identified the IS*26* and an IS*1294* surrounding the *mcr*-1 cassette in pLREC160_2.

Plasmids pLREC159_1 and pLREC176_1 identified as IncHI2-ST4 had identical genetic environments downstream the *mcr*-1 cassette.

#### Statistical Analysis

The variables (*cdt*B, *cnf*1, *hly*A, virotypes, PST, and subclades) did not follow a normal distribution (Shapiro–Wilk test: *p* < 0.0001). We performed the Mann–Whitney–Wilcoxon test to assess significative differences in the distribution of features (virotype D5| subclade B7| PST9| *cnf1*| *hly*A vs. virotype D2| subclade B6| PST43| *cdt*B variables; *p* = 0.007) between ST131 isolates from piglets with diarrhea and pork meat.

## Discussion

As far as we know, there are only six reports on ST131 from swine: (I) Trobos et al. (2009) studied 68 sulfonamide-resistant *E. coli* from different hosts and identified the first swine ST131 isolate obtained from pork meat in Denmark in 2003; (II) Schink et al. (2013) detected a CTX-M-1 ST131 isolate recovered from a porcine gastrointestinal tract infection among 495 ESBL-producing *E. coli* analyzed in Germany during the years 2006–2007; (III) Herrera Estévez (2015) detected 18 ST131 isolates from 200 pork meat samples obtained in Spain during the years 2011 and 2012; (IV) Liu X. et al. (2018) collected an isolate from a diarrheal pig among 44 ESBL-producing *E. coli* from Northwest China (2015–2017); (V) García-Meniño et al. (2018) identified 18 ST131 isolates recovered from the 1823 piglets with diarrhea sampled in Spain between the years 2009 and 2017; and (VI) Reid et al. (2019) reported two highly related ST131 *E. coli* isolates, the first from a healthy pig and the second from a human urinary tract infection, obtained in New South Wales (Australia) in 2007 and 2009, respectively.

In the present study, we performed WGS analysis of a collection of 11 representative ST131 isolates obtained from piglets with diarrhea and pork meat in Spain. All of the 11 ST131 isolates belonged to serotype O25b:H4 and clade B and presented a high number of virulence genes typical of *E. coli* that cause extraintestinal infections. In addition, the 11 porcine isolates harbored traits that genetically satisfied the criteria for designation as ExPEC, UPEC, and APEC status being designated as APEC-, UPEC-, and ExPEC-like isolates because of their site of isolation. Although seven of the 11 ST131 isolates were isolated from piglets with diarrhea, none of them were positive for virulence genes of intestinal pathogenic *E. coli* (Mora et al., 2011).

In Supplementary Figure S18, based on the results published by Matsumura et al. (2017) and Ben Zakour et al. (2016), we had represented the already known ST131 lineages plus the new lineages found in this study. These sublineages were designated according to their *fimH* allele (type 1 fimbriae adhesin gene), phylogenetic clade (A, B, B0, I1, C0, C, C1, C2, and C3), and resistance profile. Briefly, B-*H*22 clade, a fluoroquinolone (FQ)– and cephalosporin-susceptible ancestor, evolved in C-*H*30R (where “R” indicates resistance to FQ) clade that was the predecessor of two sister subclades, C1-*H*30R and C2-*H*30Rx (often carrier of the *bla*_*CTX*__–__*M*__–__15_ enzyme). Later, two additional ST131 sublineages referred to C1-M27 (positive for the *bla*_*CTX*__–__*M*__–__27_ enzyme) and C1-nM27 (non-ESBL or positive for the *bla*_*CTX*__–__*M*__–__14_ enzyme) have been described. The ST131 clonal group accounted for 490 (16%) of 2,995 isolates obtained from extraintestinal clinical samples collected in five Spanish hospitals during the period 2005 to 2012 (Dahbi et al., 2014). The most common lineages in this clinical collection were the C2-*H*30Rx (61.6%), B (17%), and C1-nM27 (16%). Recently, Mamani et al. (2019) also described the subclade C2-*H*30Rx as the most prevalent (85%) among 41 ST131 ESBL-producing *E. coli* causing bacteremia in a Spanish hospital over a 12-year period (2000–2011). In contrast, almost all Spanish ST131 isolates obtained from animal origin belonged to clade B. In our laboratory, we have tested a collection of 98 ST131 isolates from animals (57 from avian, 36 porcine, and five from Antarctic pinnipeds), and all but one from pinnipeds belonged to clade B. Although clade B isolates share numerous virulence genes, virotypes vary depending on the origin of the isolates. Thus, all avian isolates have virotype D4 (*ibeA*, neuC-K1), whereas swine isolates have virotypes D2 (*ibeA*, *papG* III, *cdtB*, *kpsM*-II-K5) and D5 (*ibeA*, *papG* III, *cnf1*, *hlyA*, *kpsM*-II-K5). Among the 45 clade B isolates causing bacteremia in humans in Spain, the most frequent virotype was D2 (*n* = 19), followed by virotypes D3 (*n* = 12), D1 (*n* = 8), D4 (*n* = 6), and D5 (*n* = 6) (Blanco et al. unpublished data).

The CG SNP-based phylogenetic tree of 84 human and animal ST131 isolates analyzed in this study showed (Figure 1) that they are mostly clustered into three well-defined clades (A, B, and C), and similarly to Petty et al. (2014) study, we have observed that clade A is the most divergent from clades B and C. Our results also agree with those published by other authors (Ben Zakour et al., 2016; Matsumura et al., 2017; Liu C. M. et al., 2018; Decano and Downing, 2019) regarding that clade B isolates are displayed into multiple subclades. We identified the six subclades (B0, B1, B2, B3, B4, and B5) described by Ben Zakour et al. (2016) and five new ones (B6, B6-like, B7, B8, and B9). Notably, the 11 ST131 porcine isolates from this study were grouped in the new subclades B6 (four isolates from pork meat and virotype D2) and B7 (seven isolates from piglets with diarrhea and virotype D5). The new subclade B6-like also includes two isolates of porcine origin, whereas the new subclades B8 and B9 included only ST131 isolates from Antarctic pinnipeds. In contrast, avian isolates of virotype D4 were distributed in the subclades B2, B3, and B5.

The newly described B6 and B7 subclades were primarily associated with porcine isolates, but also include some isolates causing bacteremia and other extraintestinal infections in humans. Therefore, it is remarkable how those human isolates were closely related (46–60 SNPs average distance) with porcine isolates. Those results suggest the ecological overlap of human and animal isolates. Recently, Reid et al. (2019) compared the genomes of two ST131 clade B *fimH22* isolates obtained in Australia, the first one isolated in 2007 from a healthy piglet and the second one obtained in 2009 from a human urinary tract infection. This study showed that the two isolates were highly related, separated by only 20 core SNPs (Reid et al., 2019). Thus, the analysis of the phylogeny confirmed *E. coli* ST131 as pathogen capable of frequent interspecies movements.

The plasmidome of *E. coli* ST131 clade B from porcine origin had shown variability and some interesting differences concerning the actual published data. IncF plasmids are a common feature in all ST131 isolates (Lanza et al., 2014), having at least one per isolate in this study. Specifically, F1:A2:B20 and F2:A1:B- plasmids are more commonly associated with the C1-*H*30R clade and C2-*H*30Rx, respectively (Johnson et al., 2016). In contrast, F2:A-:B1 plasmids were dominant in the porcine clade B isolates used in this study. This type of plasmid was absent in 35 non-ST131 *E. coli* porcine isolates from colibacillosis (García-Meniño et al., 2019), suggesting that IncF plasmids vary greatly within the different lineages. Reid et al. (2019) reported 23 F-types among 282 isolates from a mixture of origins belonging to the clade B of ST131, and the F2:A-:B1 IncF subtype was one of the commonest (32%).

In accordance with previous studies, plasmids carrying virulence genes belong to the IncF family (Lanza et al., 2014; Nicolas-Chanoine et al., 2017). All the IncF plasmids found in this study conserved the ColV region, frequently detected among avian pathogenic *E. coli* (APEC) (Liu C. M. et al., 2018). This region comprises the *iss* (increased serum survival) gene, the *iroBCDEN* gene cluster (encoding the salmochelin siderophore system present in *Salmonella enterica* spp. and some ExPEC isolates), the *iucABCD* operon (encoding the enzymes of the biosynthetic pathway for aerobactin), *iutA* (outer membrane receptor of ferri-aerobactin complexes) gene, *cvaB* and *cvaC* (production of colicin) genes, *sitC* (*Salmonella* iron transporter C), and an *ompT-hlyF-mig14* cassette (outer membrane related genes). Some APECs cause human infection (Liu C. M. et al., 2018); nevertheless, ColV plamids were absent in specific human clades, so they may not be a necessary feature to cause human infection (Reid et al., 2019). It is currently unknown which advantages could the IncF plasmids confer to the bacterial cell apart from virulence and resistance to antibiotics. However, Johnson et al. (2016) has hypothesized that they must play multiple roles in the host success because ST131 clone has adapted to harbor these plasmids at lower fitness cost and has stabilized them through mutational events. We assume that the presence of both, resistance and virulence genes, on the same MGE is worrying for public health, as shown in our study in IncF plasmids such as pLREC161_1 and pLREC 159_2. For instance, the evolutionary analysis performed by Ben Zakour et al. (2016) proved that ST131 emergence was adapted by isolates that had acquired first a subset of virulence genes followed by the gain of antibiotic resistances.

ColE1-like plasmids were the second more prevalent in our collection and the replication protein could not be determined; these results are in accordance with the findings of Lanza et al. (2014). Furthermore, ColE plasmids have been reported as *mcr-4* and *mcr-5* carriers (Borowiak et al., 2017; Carattoli et al., 2017; García-Meniño et al., 2019). The ColE1-like plasmids from this study did not carry resistance genes. However, they might be an important adaptive weapon. Moreover, we described one MOB_P__131_ and two MOB_V__2_ plasmids, which are not frequently documented and therefore absent among the description given by Lanza et al. (2014) over ST131 plasmidome, although another MOB_P__131_ plasmid has been recently found from a bla_OXA–__48_ *E. coli* ST131 isolate collected from a patient with urinary infection (Stoesser et al., 2016c), and MOB_V__2_ plasmids have been described in other STs, such as ST69 from a human urine sample and ST93 from chicken meat samples (de Been et al., 2014). Besides, MOB_V__2_ plasmids pLREC153_4 and pLREC157_4 showed structural homology (around coordinates 3.7–7 kb in Supplementary Figure S9) with the pEC0674 plasmid (6 kb), an *mcr-5* carrier plasmid belonging from an ST29 *E. coli* isolate recovered from porcine feces in Germany (Hammerl et al., 2018). Finally, and also barely documented, were the cryptic plasmids found in our study. Similar small no-MOB plasmids such as pLREC158_3, pLREC159_5, pLREC159_6, pLREC160_7, and pLREC161_4 have been reported among ST131 isolates. The role of these plasmids is still unknown, but researchers have suggested that they could be providing useful mobilization tools for ST131 adaptation (Lanza et al., 2014).

Antimicrobial resistance genes conferring resistance to penicillin/ampicillin, tetracycline, quinolones, and colistin were the most common determinants. García-Meniño et al. (2019) described chromosomal mutation (PmrB V161G or PmrA S39I) in addition to the plasmid mechanism (*mcr*) in 16 (46%) of non-ST131 colistin-resistant porcine *E. coli* isolates. Here, any complementary chromosomal mechanism related with system PmrAB was found. Nonetheless, some drug efflux genes were found in plasmids. Members of the *lnu* (previously *lin*) gene family (mediate lincosamide resistance) (Achard et al., 2005; Roberts, 2008) and the *mef*(*B*) gene, which coexists with the *aadA* and *sul3* resistance genes (pLREC161_2). This combination has previously been described in plasmids from porcine *E. coli* isolates and mediates macrolide resistance (Liu et al., 2009).

The recent analysis of 35 colistin-resistant non-ST131 *E. coli* isolates from porcine colibacillosis showed that 18 isolates (51%) were carriers of the *mcr-*1.1 gene variant. Those isolates were all MDR, and the 50% belonged to Clonal Complex 10 (6 ST10, 1 ST5786, and 2 ST7323). However, the remaining isolates belonged to different linages with single representants (ST1, ST29, ST42, ST93, ST100, ST118, ST156, ST398, and ST4247). The *mcr*-1.1 gene was located in plasmids IncHI2 (33%) and IncX4 (22%), but it was also found chromosomally encoded in four isolates (García-Meniño et al., 2019).

Currently, the *mcr*-1 cassette was found in a large diversity of plasmids and clonal lineages of *E. coli*. The broad adaptation of colistin resistance all over the world could be due to the ease with which the *mcr*-1 gene integrates into various regions (Hadjadj et al., 2017). There is an association with successful plasmid families (Matamoros et al., 2017; Zhong et al., 2018). On the other hand, any association with clonal lineages of *E. coli* is described (Li et al., 2018). However, the description of *mcr*-1–positive *E. coli* ST131 isolates is exceptionally worrying. Because ST131 has once been responsible for the worldwide appearance, ESBL resistances due to the acquisition of specific IncF epidemic plasmids harboring *bla*_CTX–M–__15_ gene (Nicolas-Chanoine et al., 2008, 2014; Mathers et al., 2015) and therefore major therapeutic failure could come due to the association of *mcr*-1 gene with other broad-spectrum resistance mechanisms, such as ESBLs and/or carbapenemases (Du et al., 2016; Haenni et al., 2016; Li et al., 2016; Olaitan et al., 2016; Skov and Monnet, 2016). Further, clade B of ST131 has been signaled out as a potential foodborne uropathogen and as a stronger early biofilm producer than clade C of ST131 (Nicolas-Chanoine et al., 2017; Liu C. M. et al., 2018; Flament-Simon et al., 2019).

In this study, the genetic context of *mcr*-1 gene was diverse. Recent studies propose that the transposon Tn6330, flanked at both ends by ISA*pl1*, has led the mobilization of *mcr*-1 gene. Therefore, four structures of mobile elements carrying *mcr*-1 have been described: (I) the composite transposon Tn6330 (ISA*pl1*–*mcr*-1–orf–ISA*pl1* structure); (II) a single ISA*pl1* upstream (ISA*pl1*–*mcr*-1–orf structure); (III) a single ISA*pl1* downstream (*mcr*-1–orf–ISA*pl1* structure); and (IV) and sequences lacking ISA*pl1* altogether (*mcr*-1–orf structure), although a few other truncated or interrupted versions have been reported (Pham Thanh et al., 2016; Snesrud et al., 2016). In the study carried by Snesrud et al. (2018), prevalence rates of 11% for structure I, 22% for structure II, 1% for structure III, and 66% for structure IV (with no associated IS) have been described. Besides, the upstream ISA*pl1* element was observed in 78% of IncHI2 plasmids (Matamoros et al., 2017), whereas the *mcr*-1–orf structure has been described as dominant in small *mcr*-1–IncX4 plasmids (Li et al., 2017b; Sun et al., 2017). These results are in line with our findings. The five ST131 colistin-resistant isolates from this study presented the *mcr*-1.1 allele (KP347127.1) and showed the *mcr*-1–orf structure. However, pLREC159_1 (IncHI2) presumptively harbored the ISA*pl1* upstream, whereas any copy of ISApl1 was present around the *mcr*-1 gene in pLREC160_2 (IncX4). Nevertheless, pap2 gene and ISs IS26 and IS1924 were present in pLREC160_2. Zhong et al. (2018) suggested that for IncX4 plasmids IS26/26 structures could be involved in *mcr*-1 gene mobilization. The BRIG comparison showed that the *mcr*-1–harboring IncX4 plasmids were genetically homologous between each other as previously described (Sun et al., 2017), suggesting that mobilization of the *mcr-*1 cassette was quite stable within this type of plasmids. Furthermore, pLREC159_1 and pLREC176_1 were almost identical in structure around the *mcr*-1 gene and were related to the presence of tellurium resistance genes, as well as for pLREC161_1. This heavy metal resistance lends HI2 plasmids to success in wastewater environments (Reid et al., 2019).

Moreover, a highly relevant finding of the present study was the appearance of chromosomally encoded colistin resistance. We found a presumptive ISA*pl1*–*mcr*-1–orf–ISA*pl1*-like structure. This genetic arrangement was described as the most prevalent by Li et al. (2018) in bacterial chromosomes. It has been proposed that the lack of one or both of the ISA*pl1*-elements implies stabilization of the *mcr*-1 gene (non-transposable) (Snesrud et al., 2016, 2018). Thus, chromosomally encoded colistin resistance might mean a more stable inheritance in ST131 porcine lineage. Nevertheless, it also implies less mobilization among isolates.

Some studies have proved that stopping using colistin in positive *mcr*-1 carriers porcine farms has resulted in the disappearance of the resistance (Randall et al., 2018). However, because of the MDR profile of our ST131 isolates, this might not be that easy, due to coselection by other antimicrobials and host fitness adaptation. The fitness effects of carrying the *mcr*-1 gene were evaluated by Wu et al. (2018). They proved that IncI2 and IncX4 *mcr*-1–positive plasmids conferred fitness advantage for its host. In contrast, IncHI2 plasmids imposed a slight fitness cost and competitive disadvantage.

To sum up, it seems that epidemic plasmid types IncX4 and IncHI2, which have been reported as the more prevalent carriers of *mcr*-1 gene, are responsible for the acquisition of the colistin resistance in the high-risk clone ST131 of *E. coli* in our isolates from porcine origin. Worryingly, the *mcr*-1 gene has also integrated an IncF plasmid from ST131. The clinical significance of this category of plasmids is therefore highlighted because they are responsible for the epidemic dissemination of ESBL resistance worldwide (Johnson et al., 2016). We hypothesize that IncF plasmids such as pLREC161_1 had acquired the *mcr* gene via cointegrating an IncHI2 *mcr*-1–harboring plasmid. The *mcr*-carrying IncF plasmid pMR0516*mcr* that has been described by McGann et al. (2016) also shared 89 kb with pHNSHP45-2 (*mcr*-1–carrying IncHI2 plasmid), and possibly the same phenomenon had occurred.

The impact that humans have on the environment and other species is evident. A multidisciplinary point of view, taking into account the environment, animals, and human for the surveillance of health, is given by the concept of “One Health” (Destoumieux-Garzón et al., 2018). This philosophy fits perfectly the actual global situation. Measures to reinforce prevention and proper management for overall antimicrobials might be the right strategy to fight against antibiotic resistance.

Further studies to understand why some plasmids evolve to become genetically stable are necessary to be able to anticipate the evolutionary dynamic of high risk clones.

As a limitation, it is worth mentioning that short reads obtained by WGS do not always allow a perfect genome reconstruction due to the high amount of repeated sequences in the bacterial genomes. Long read sequencing technologies will probably bring new perspectives in future researches.

## Conclusion

Clade B of ST131 showed a huge genetic diversity, and five new subclades were defined (B6, B6-like, B7, B8, and B9). The majority of ST131 porcine isolates belong to new subclades B6 and B7. An association between the phylogeny, the virotype, and the origin of porcine isolates was established. Some porcine and human clinical isolates were highly related. Most porcine ST131 isolates are MDR (91%) and carry many ARGs. Colistin resistance was introduced through MGEs and had been able to stabilize chromosomally. IncX4 and IncHI2 epidemic *mcr-1*–harboring plasmids are responsible for acquired colistin resistance encoded by *mcr*-1.1 gene. The surrounding environment of the *mcr-*1 cassette is variable but within the same family of plasmids, insertion, and stabilization had common structures. The plasmidome of ST131 clade B is distinct from other clades within ST131 clone and contains an impressive variety of different plasmids. Characteristically ST131 porcine isolates have APEC [F2:A-:B1]–IncF plasmids. ColE1-like plasmids and IncX plasmids are also frequently observed. This behavior could be related to the clonal success due to its ability to easily rearrange DNA by horizontal gene transfer.

## Data Availability Statement

The datasets of the 11 LREC genomes for this study can be found in the NCBI sequence databases as part of BioProject SUB5714329. With accession codes SAMN11936814 to SAMN11937940. Accession in NCBI is PRJNA546088.

## Author Contributions

S-CF-S, MT, AM, VG, IG-M, DD-J, and AH undertook the laboratory work. MT and JB conceived the concept for the manuscript and designed the experiments. All authors provided the critical input and contributed to the writing of the manuscript and approved the final version.

## Conflict of Interest

The authors declare that the research was conducted in the absence of any commercial or financial relationships that could be construed as a potential conflict of interest.

## Footnotes

1 http://enterobase.warwick.ac.uk/species/index/ecoli

2 https://www.ncbi.nlm.nih.gov/bioproject/

3 http://genomicepidemiology.org/

4 https://www.ncbi.nlm.nih.gov/pubmed

5 https://www.xlstat.com
