# Supplementary material for: Whole Genome Sequencing and Characteristics of mcr-1–Harboring Plasmids of Porcine Escherichia coli Isolates Belonging to the High-Risk Clone O25b:H4-ST131 Clade B
Source: Front Microbiol. 2020 Mar 24;11:387. doi: 10.3389/fmicb.2020.00387 (PMC7105644; doi:10.3389/fmicb.2020.00387)
Supplement: Supplementary file 5 [file Data_Sheet_1.PDF]

Supplementary Material

1 Supplementary Figures

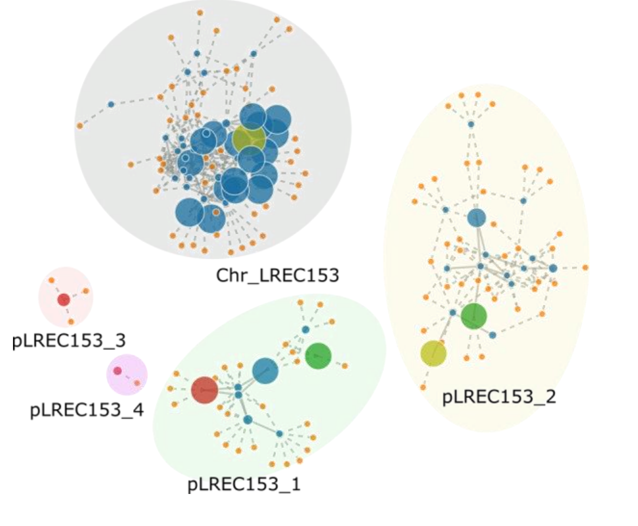

LREC_153

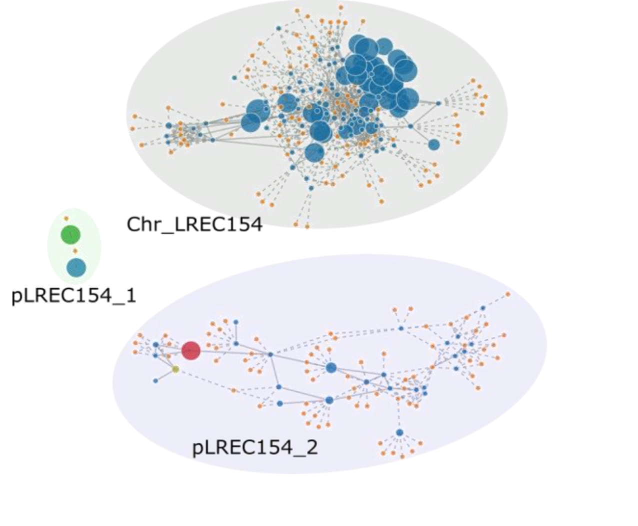

LREC_154

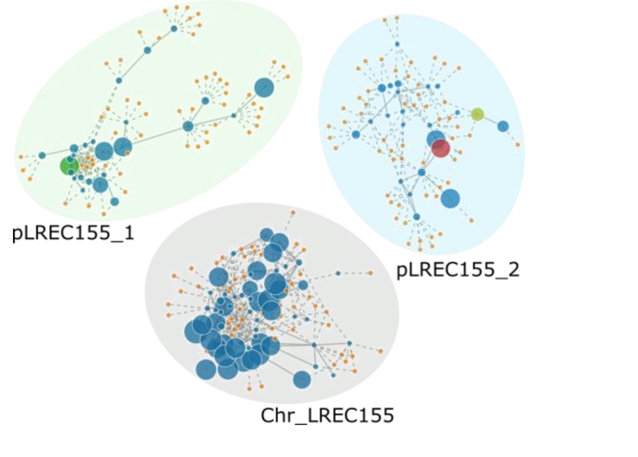

LREC_155

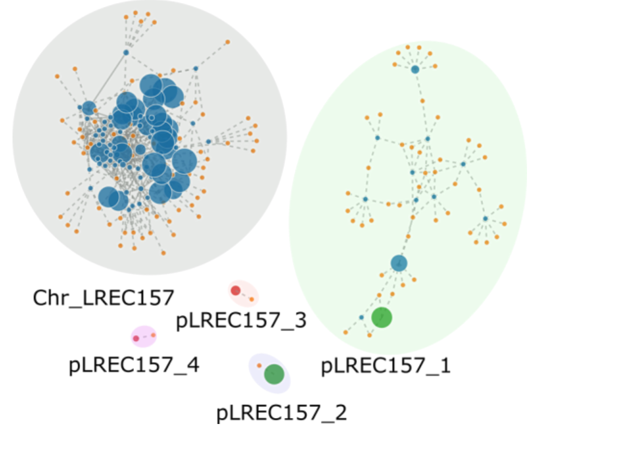

LREC_157

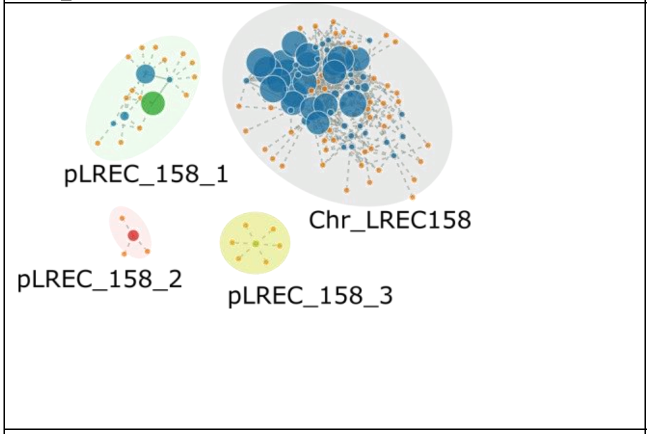

LREC_158

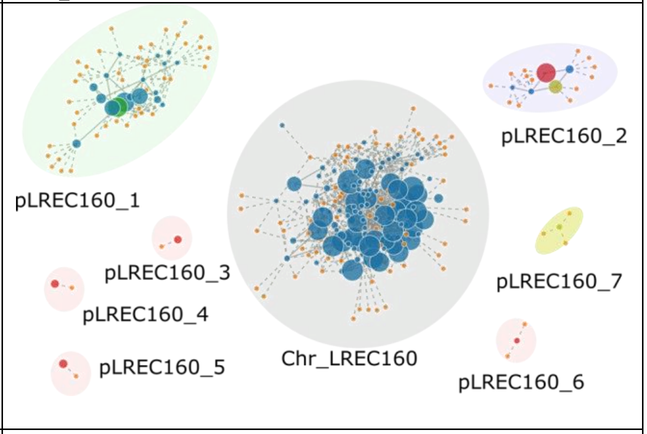

LREC_160

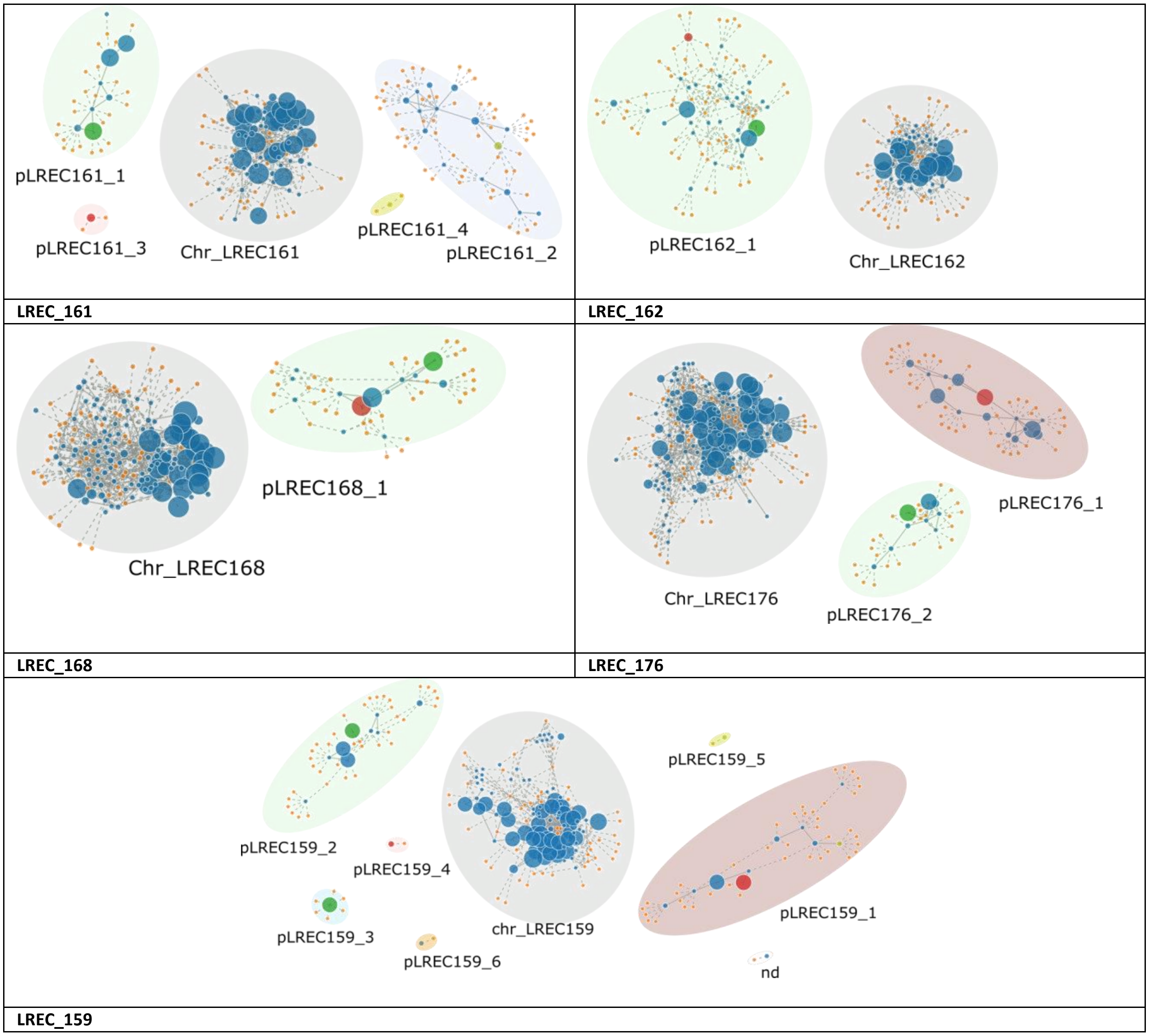

*nd, is a 2,014 pb contig (transposases + aminoglycoside resistance protein + LinF) which could not be assigned due of lack of scaffold links to both chromosome and plasmids.

Supplementary Figure 1. PLACNETw genome reconstruction of the 11 ST131 isolates from porcine origin characterized in this study. The network contains nodes of different colours (blue for contigs, orange for references, yellow for replication proteins, red for relaxases and green for replication proteins and relaxases). Nodes are linked by scaffold links (solid grey lines). The size of the contig nodes is proportional to the contig length (Lanza *et al.,* 2014).

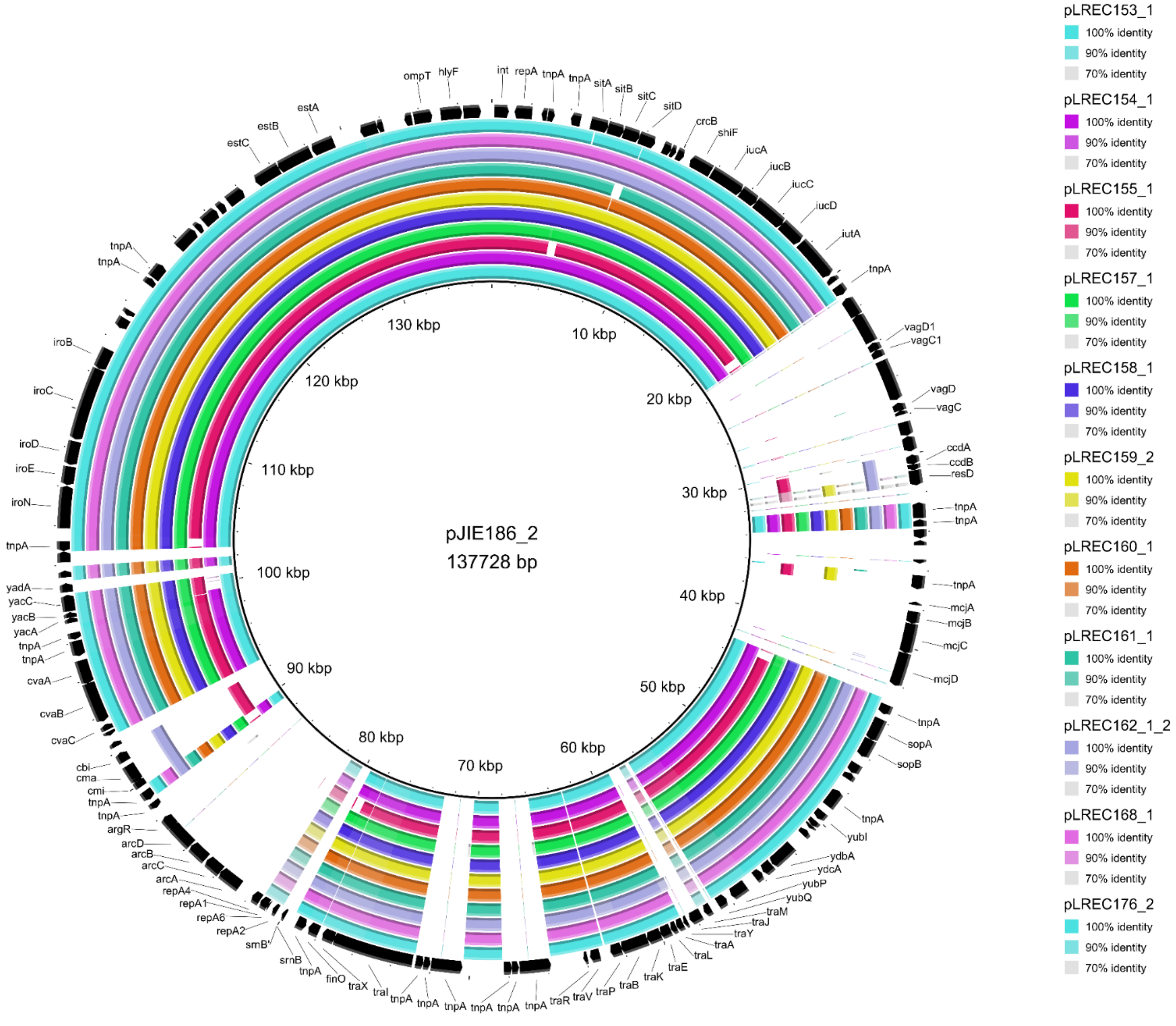

Supplementary Figure 2. Structural comparison of IncF plasmids from the 11 ST131 clade B porcine isolates.

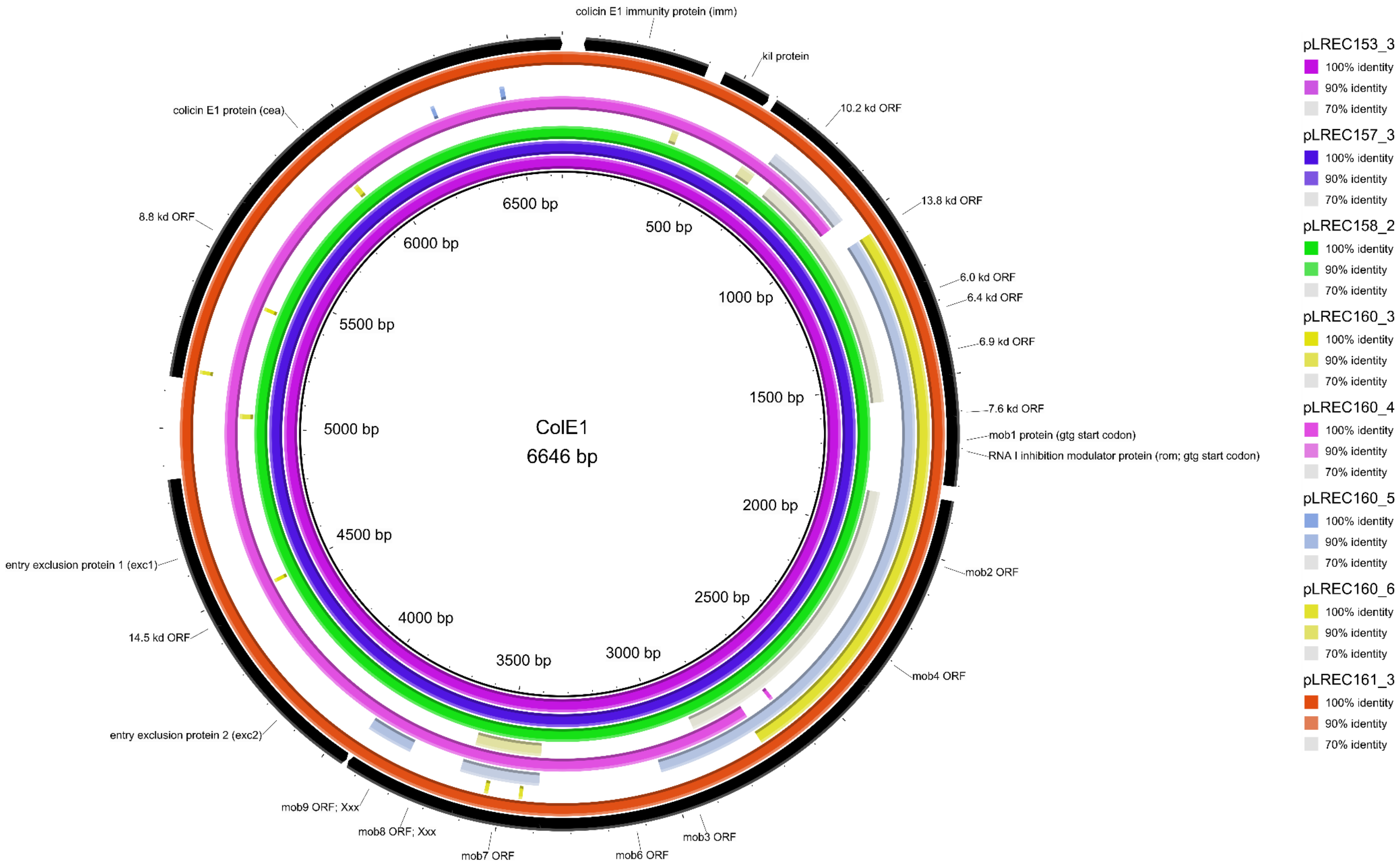

Supplementary Figure 3. Structural comparison of ColE1-like plasmids from ST131 clade B porcine isolates.

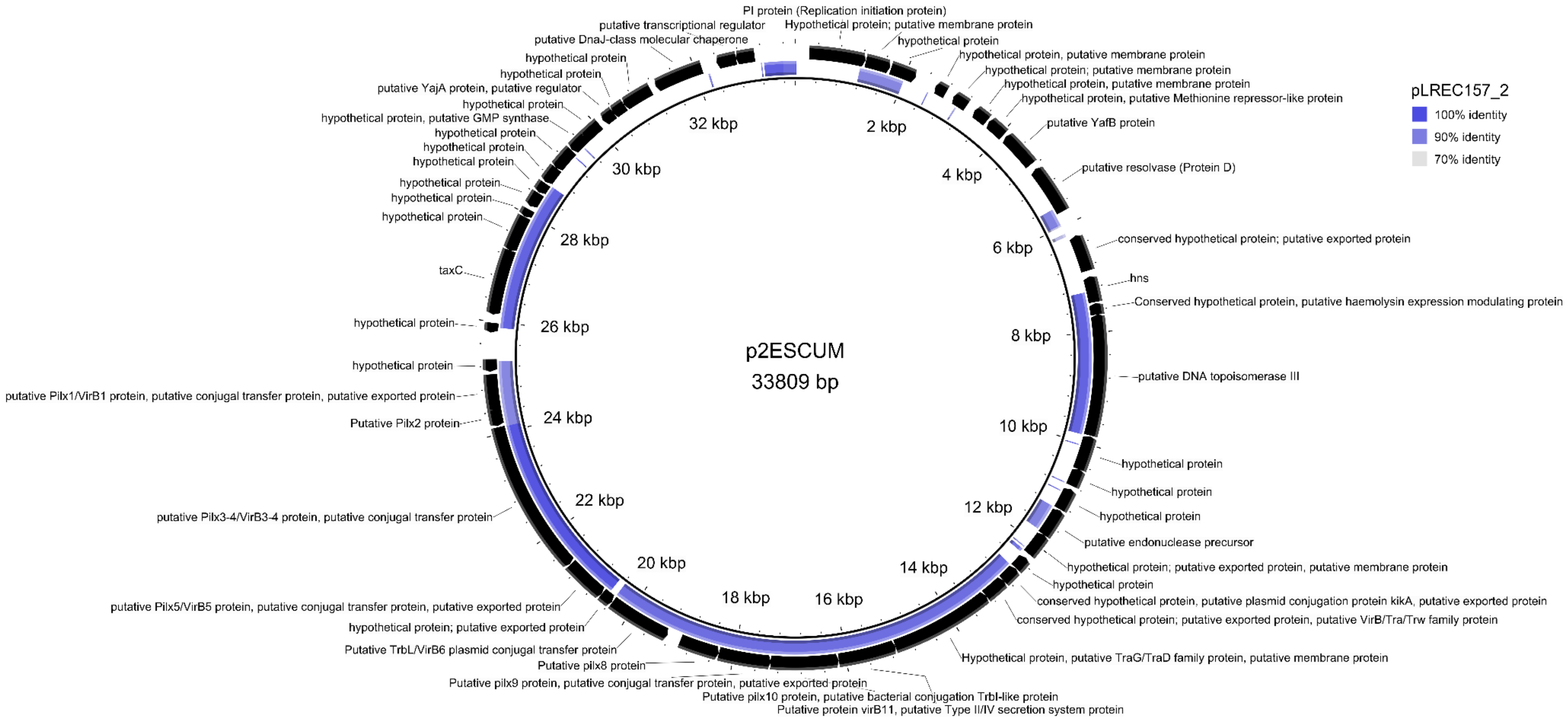

Supplementary Figure 4. Structural comparison of an IncX1 plasmid from a ST131 clade B porcine isolate with p2ESCUM as a reference.

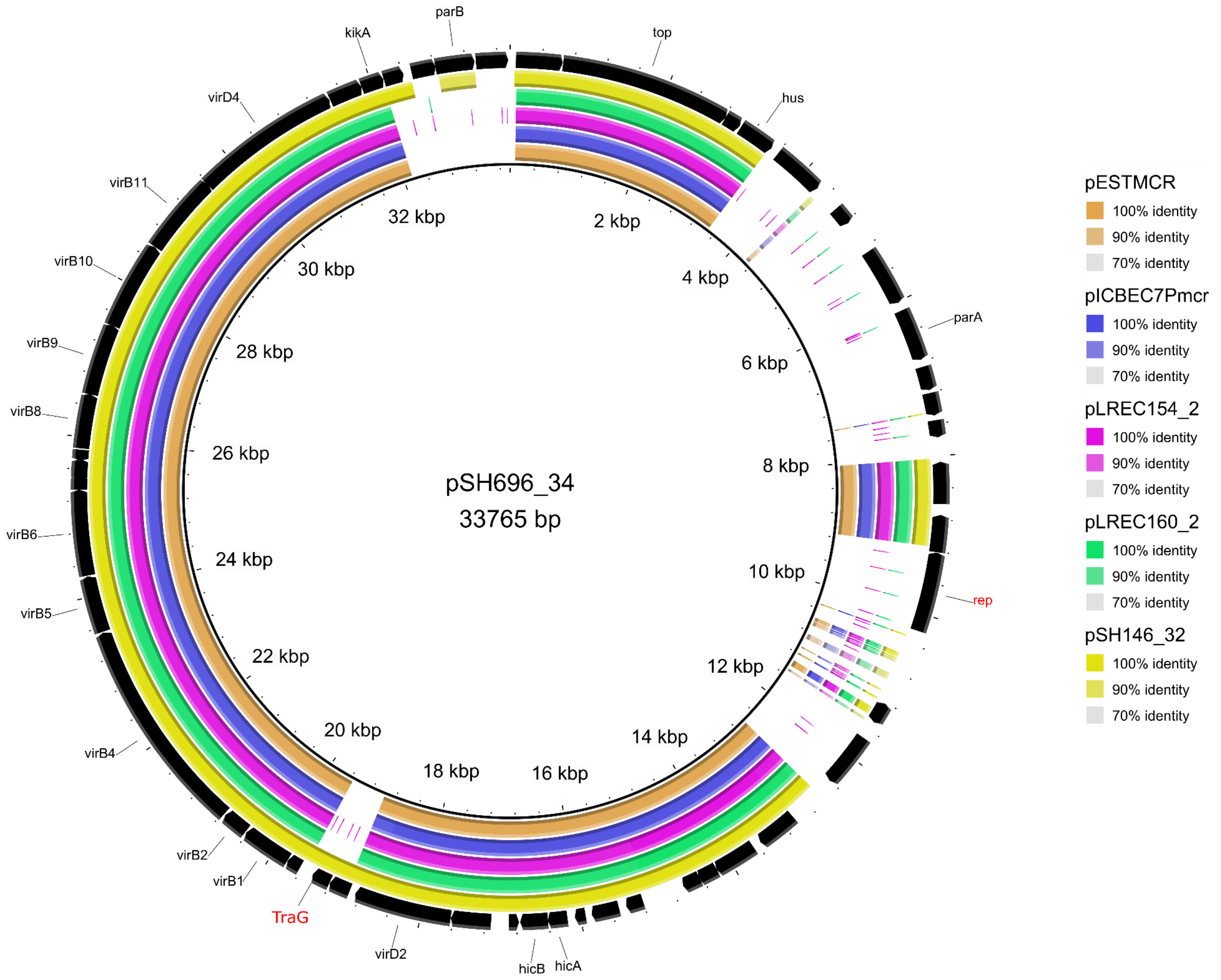

Supplementary Figure 5. Structural comparison of IncX4 plasmids from ST131 clade B isolates.

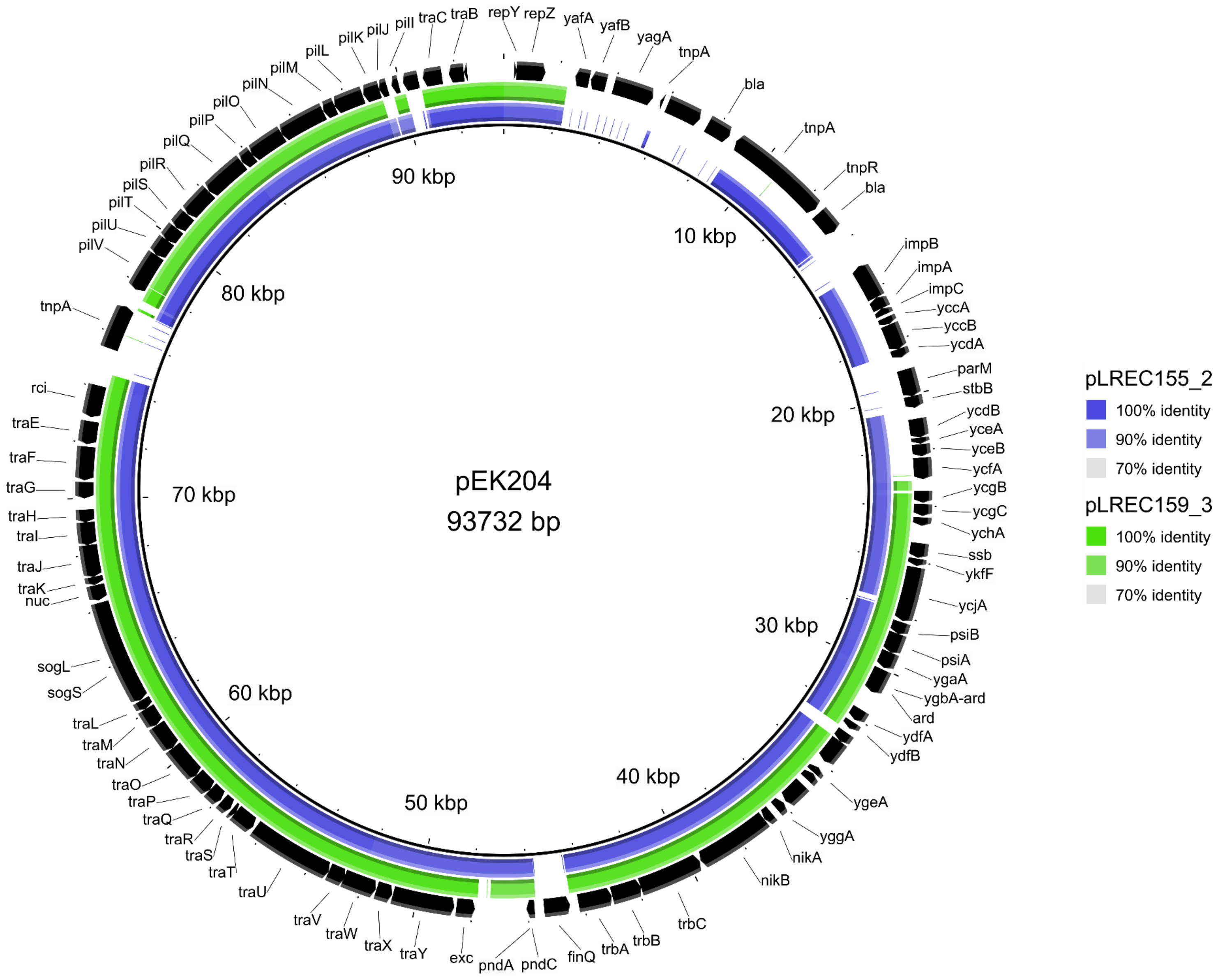

Supplementary Figure 6. Structural comparison of IncI1 plasmids from the ST131 clade B porcine isolates with pEK204 as a reference.

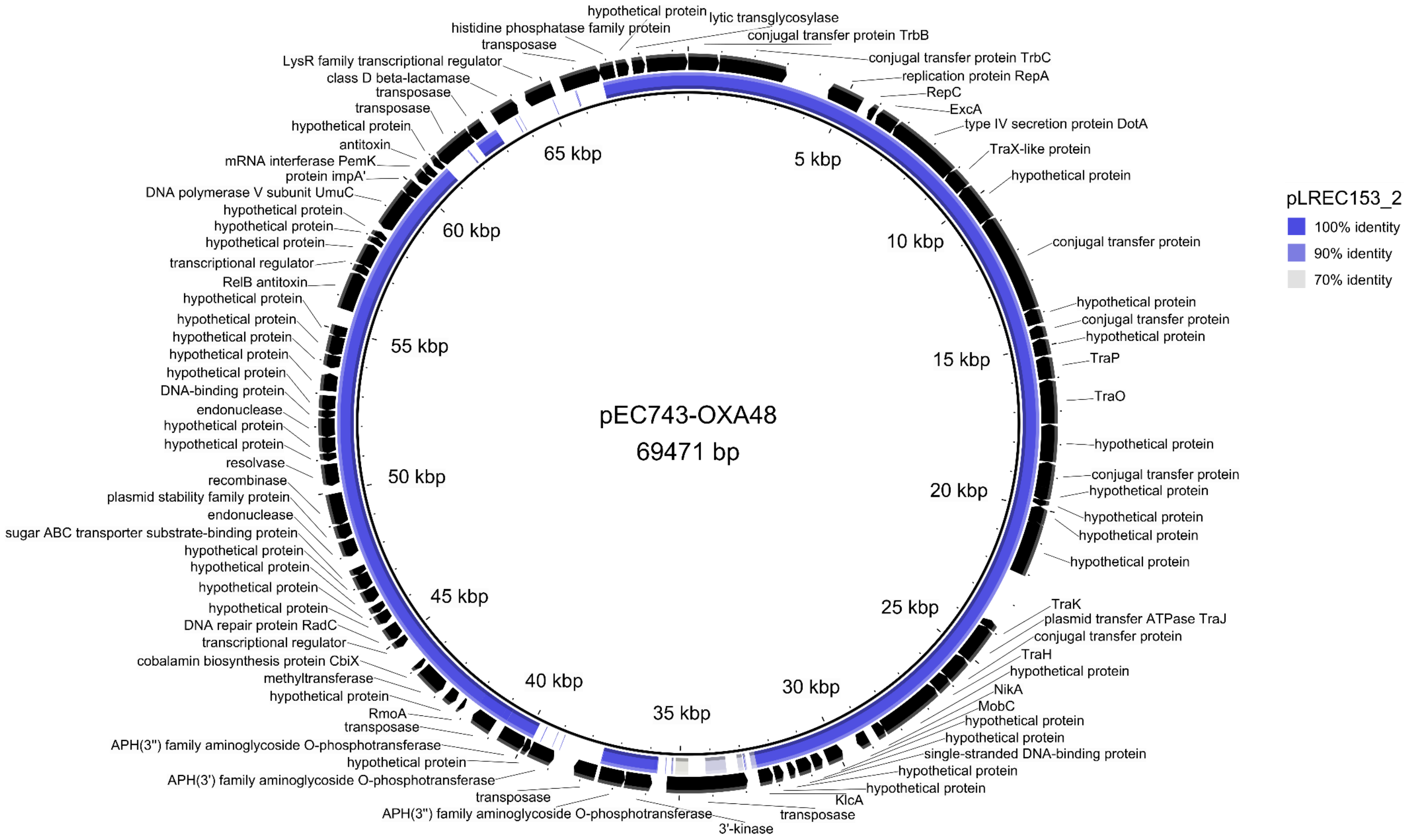

Supplementary Figure 7. Structural comparison of the IncL/M plasmid from a ST131 clade B porcine isolate with pEC743-OXA48 as a reference.

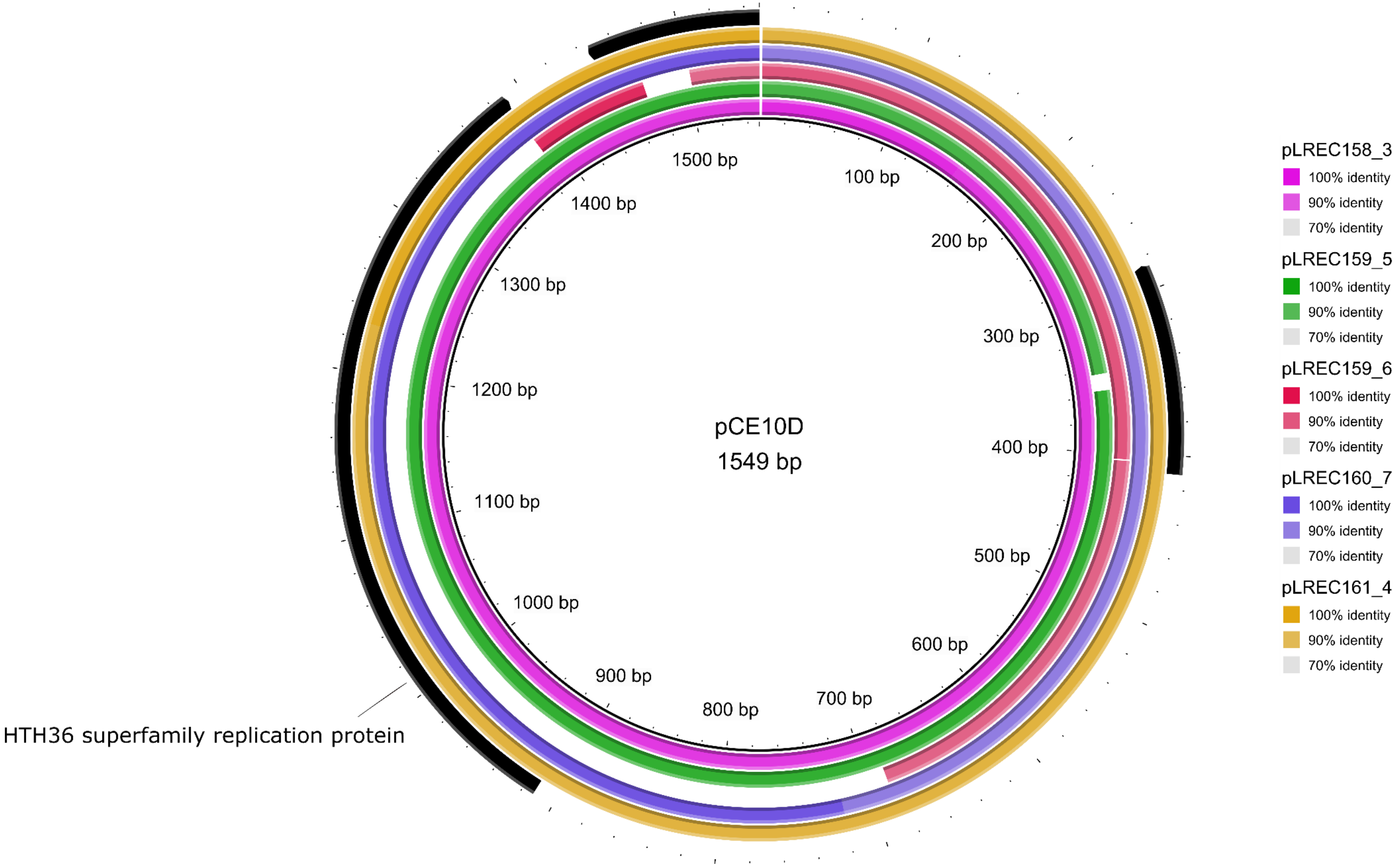

Supplementary Figure 8. Structural comparison of the cryptic no-MOB plasmids from ST131 clade B porcine isolates with pCE10D as a reference.

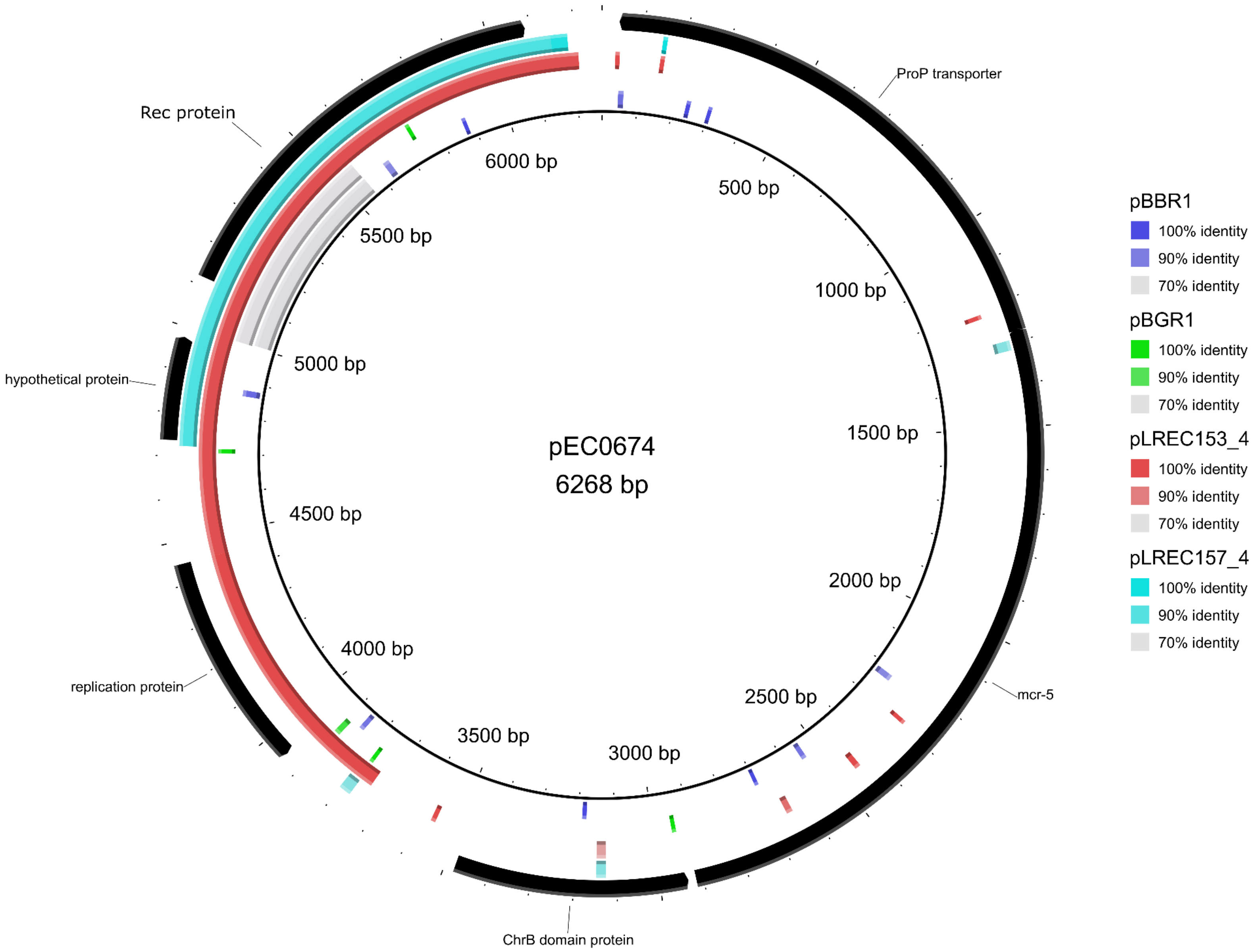

Supplementary Figure 9. Structural comparison of small MOB$_{V2}$ plasmids, ST131 clade B porcine plasmids with pEC0674 as a reference.

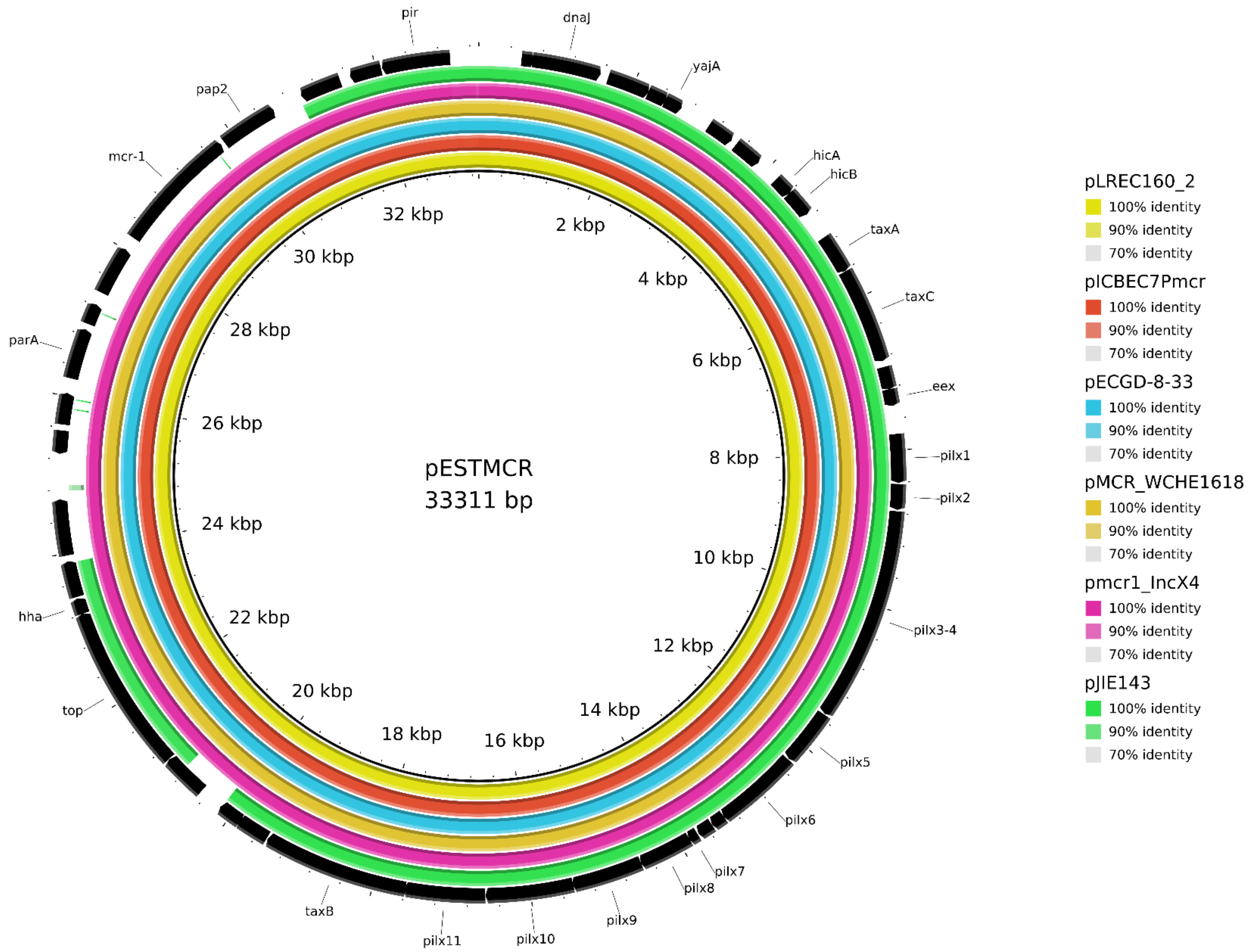

Supplementary Figure 10. Structural comparison between IncX4 *mcr*-1 and non-*mcr*-1 plasmids with pESTMCR as reference.

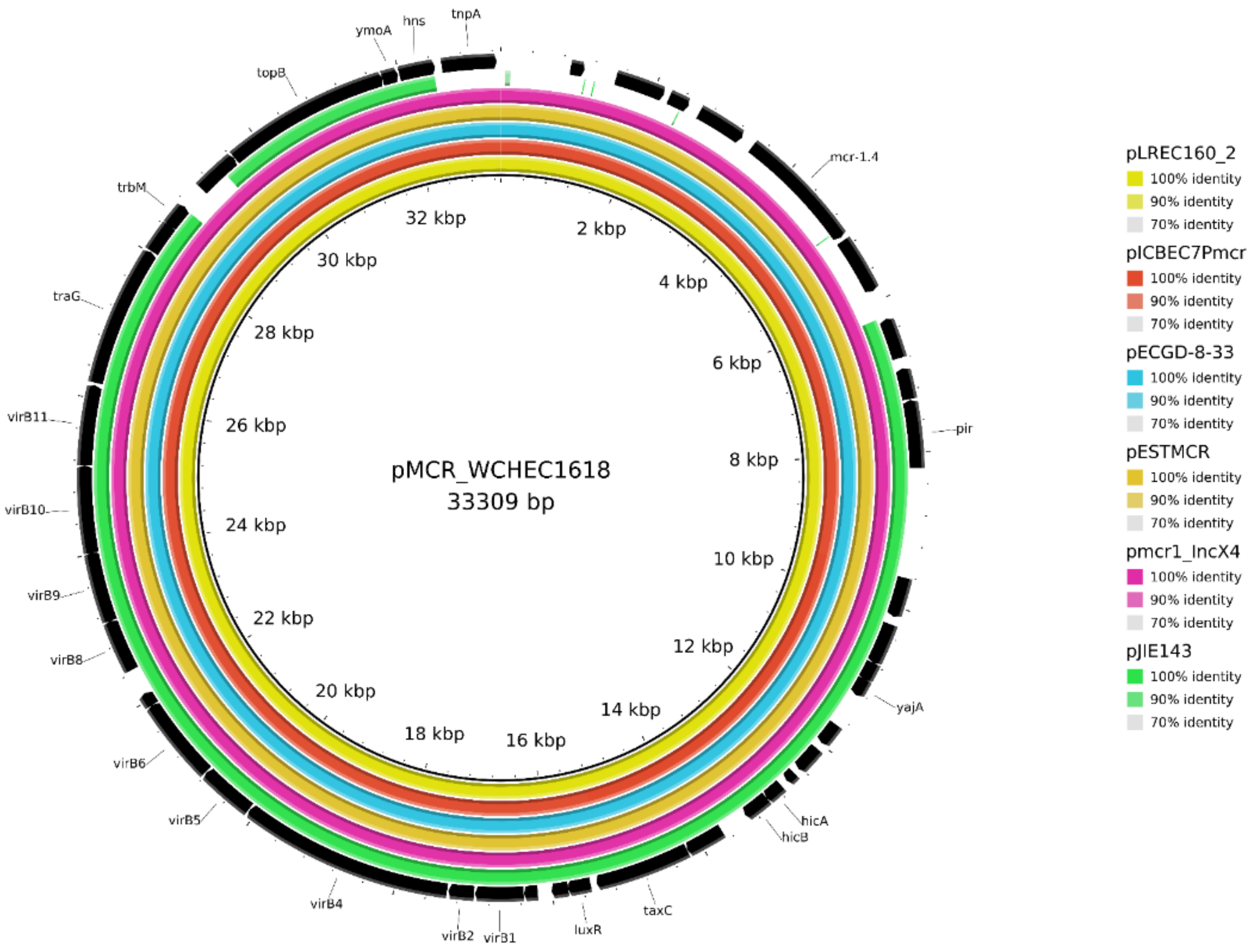

Supplementary Figure 11. Structural comparison between IncX4 *mcr*-1 and non-*mcr*-1 plasmids with pMCR_WCHEC1618 as reference.

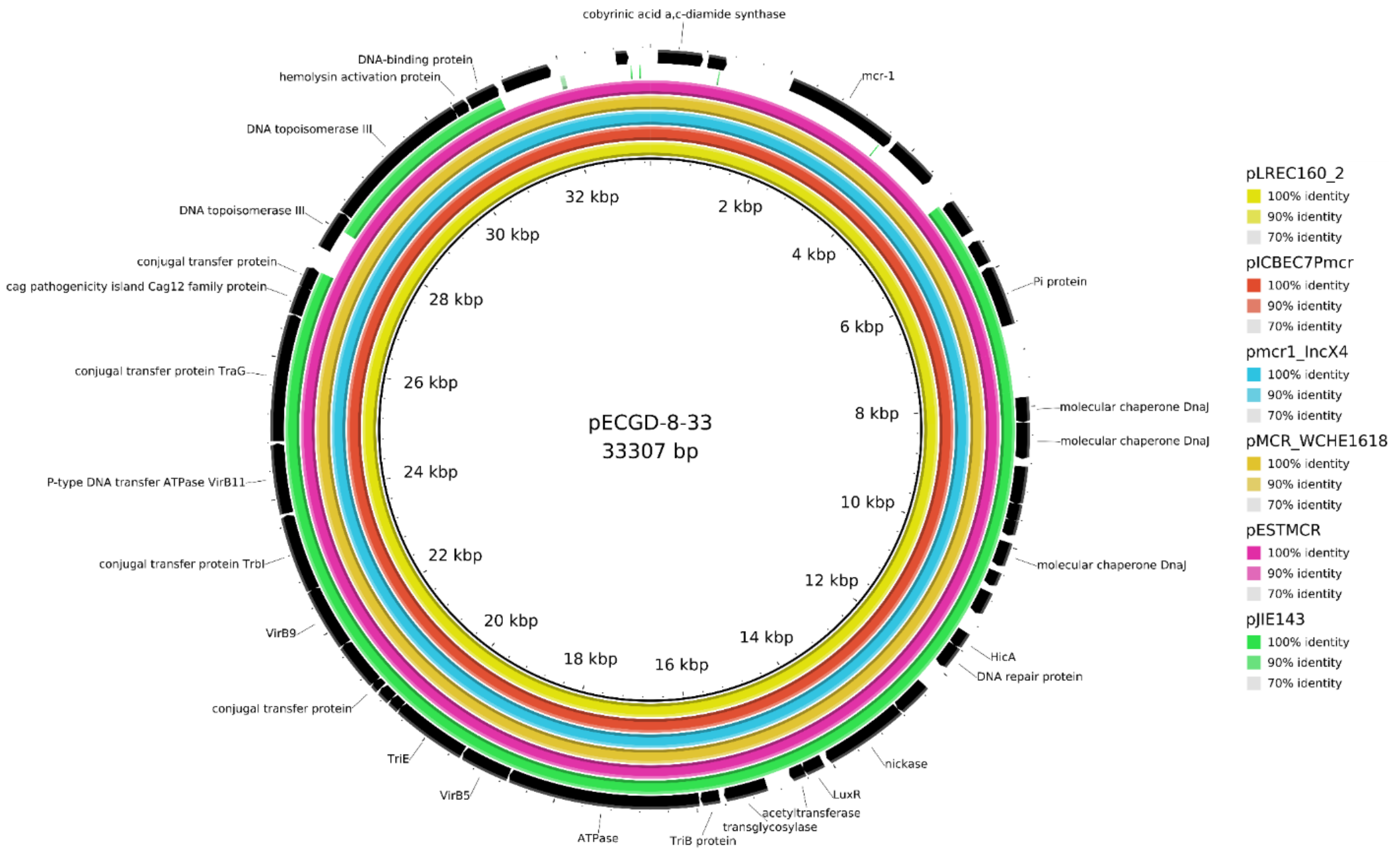

Supplementary Figure 12. Structural comparison between IncX4 *mcr*-1 and non-*mcr*-1 plasmids with pECGD-8-33 as reference.

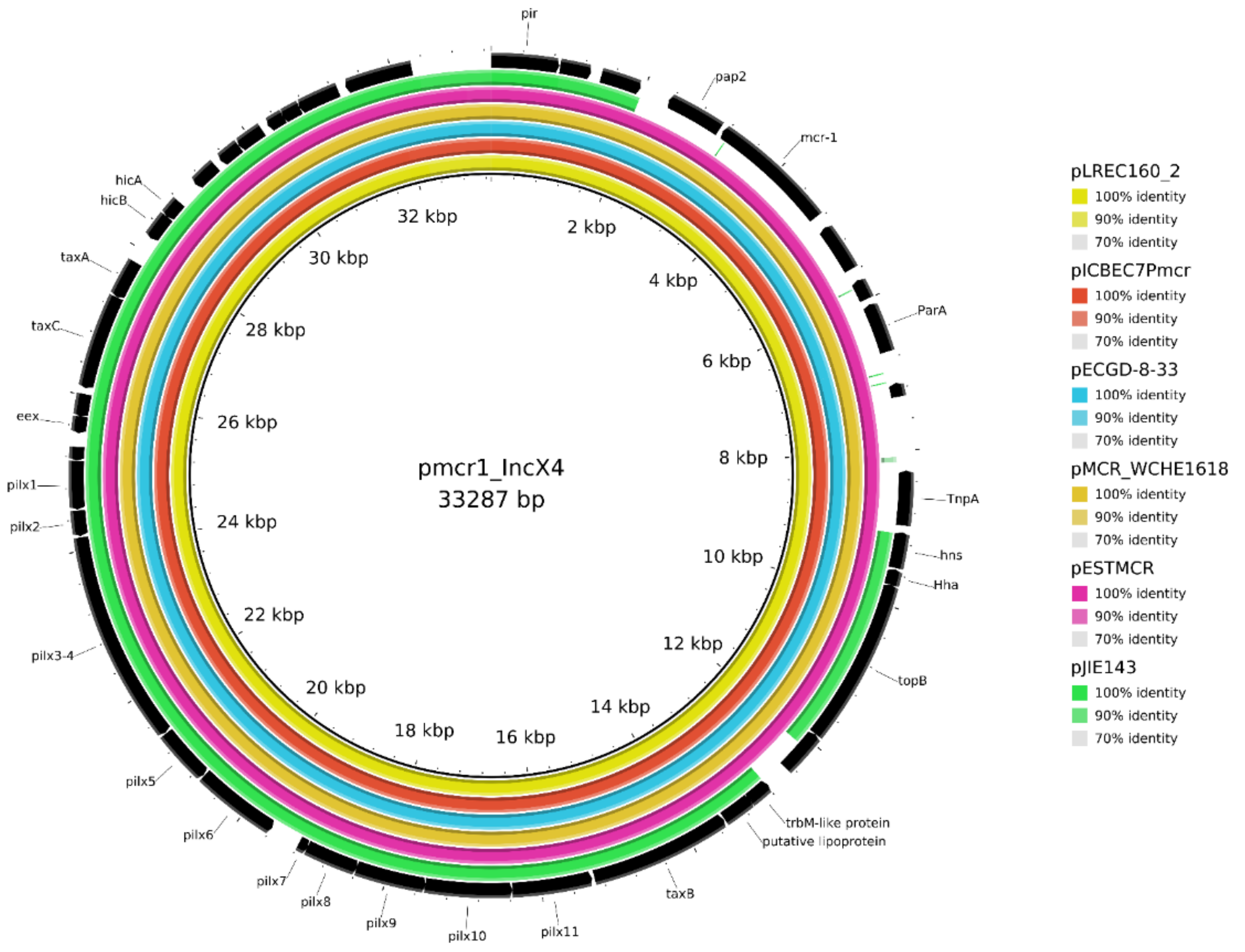

Supplementary Figure 13. Structural comparison between IncX4 *mcr*-1 and non-*mcr*-1 plasmids with pmcr1_IncX4 as reference.

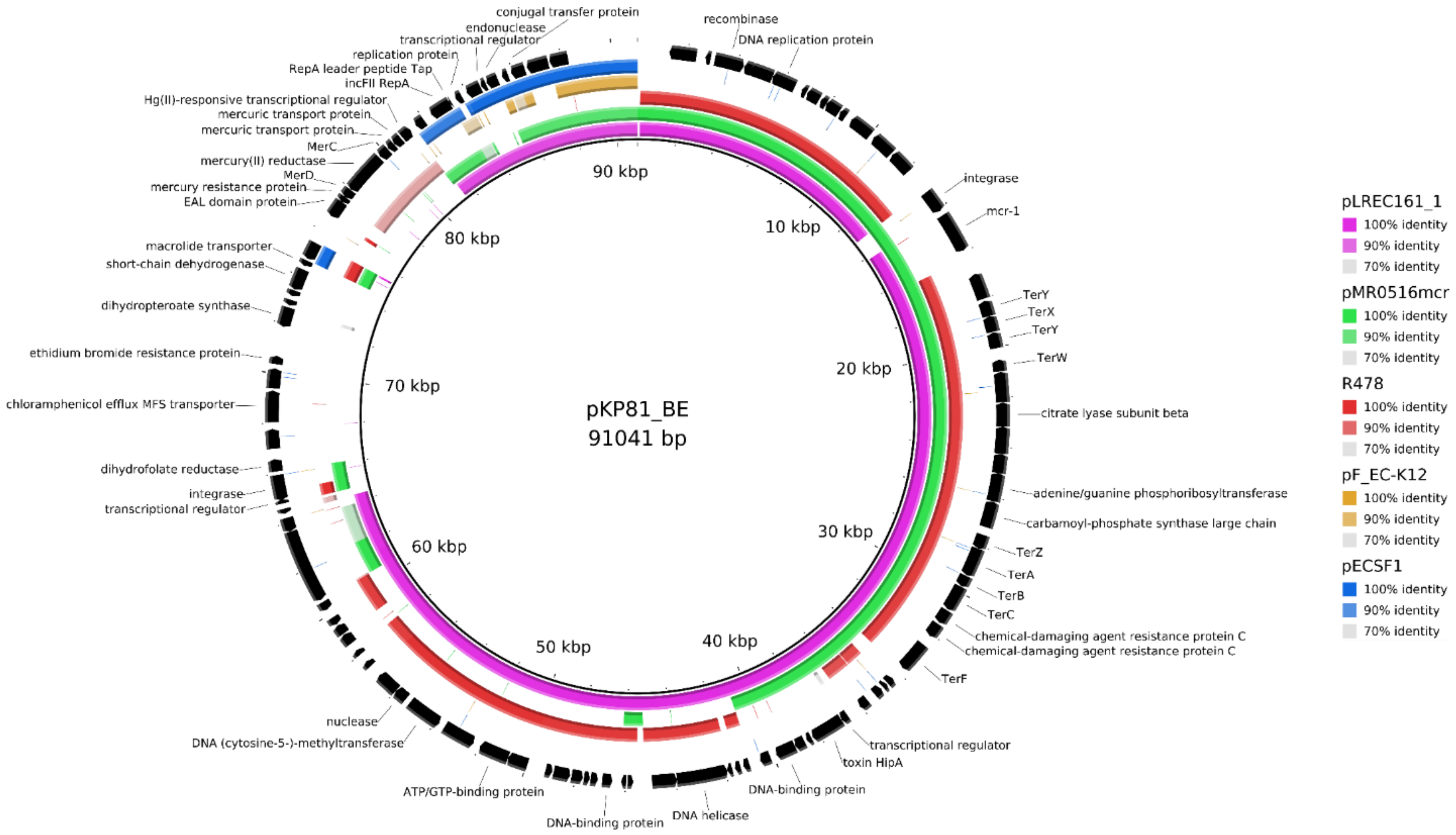

Supplementary Figure 14. Structural comparison between IncF *mcr*-1 and non-*mcr*-1 plasmids with pKP81_BE as reference.

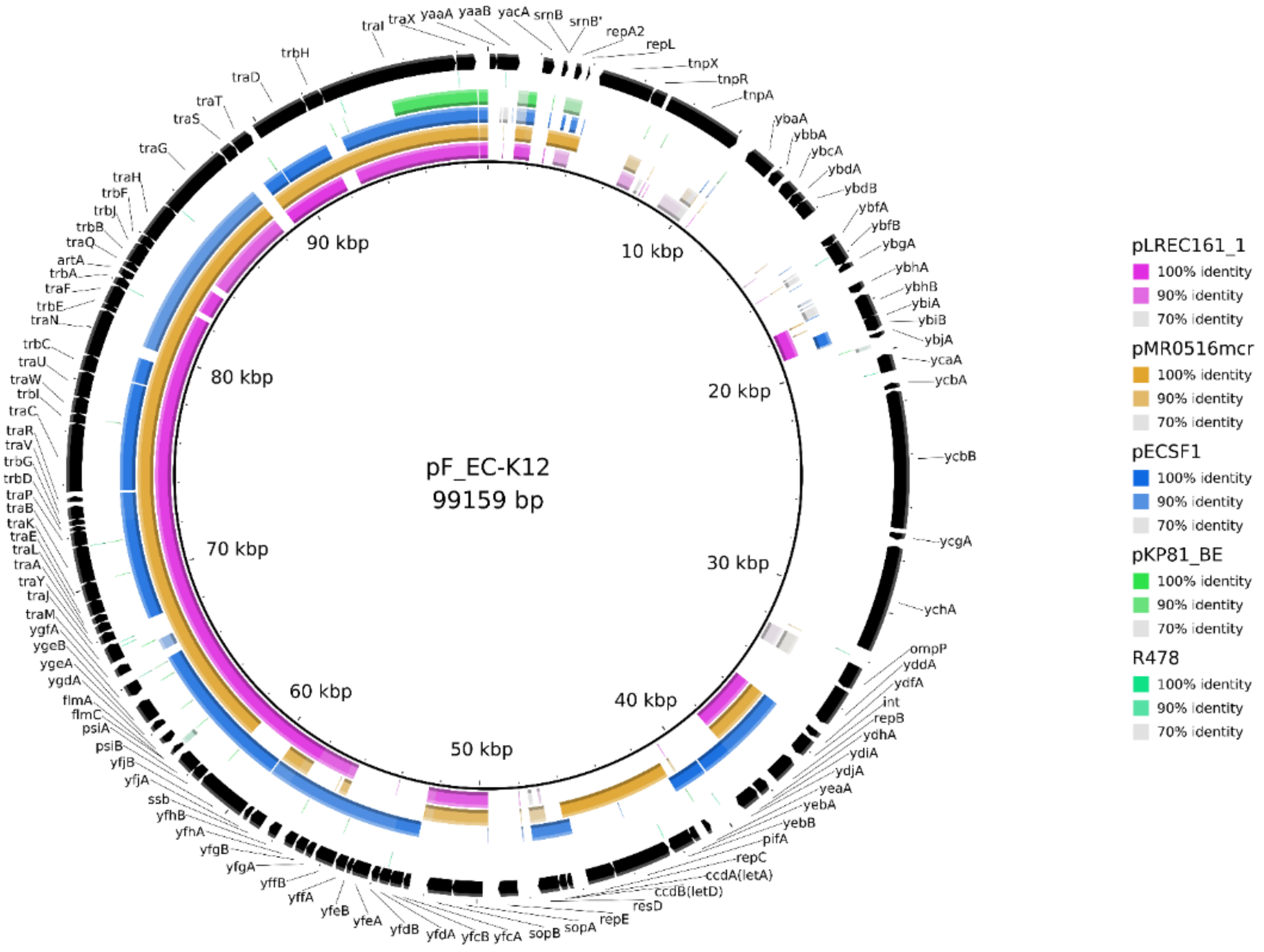

Supplementary Figure 15. Structural comparison between IncF *mcr*-1 and non-*mcr*-1 plasmids with plasmid F from *Escherichia coli* K-12 (pF_EC-K12) as reference.

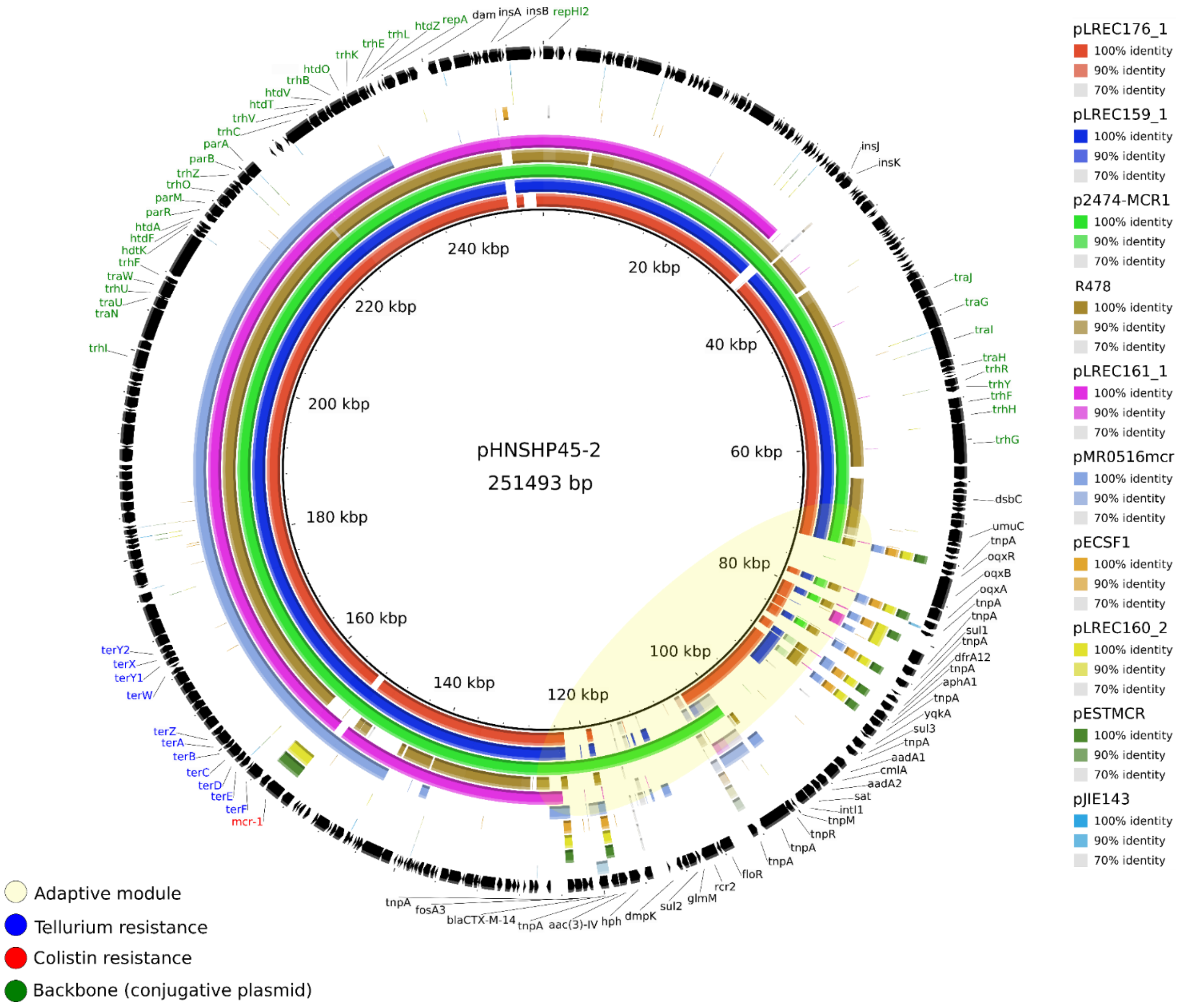

Supplementary Figure 16. Structural comparison between plasmids from ST131 *mcr*-1 porcine isolates, other *mcr*-1 plasmids and non-*mcr*-1 plasmids with pHNSHP45-2 as reference.

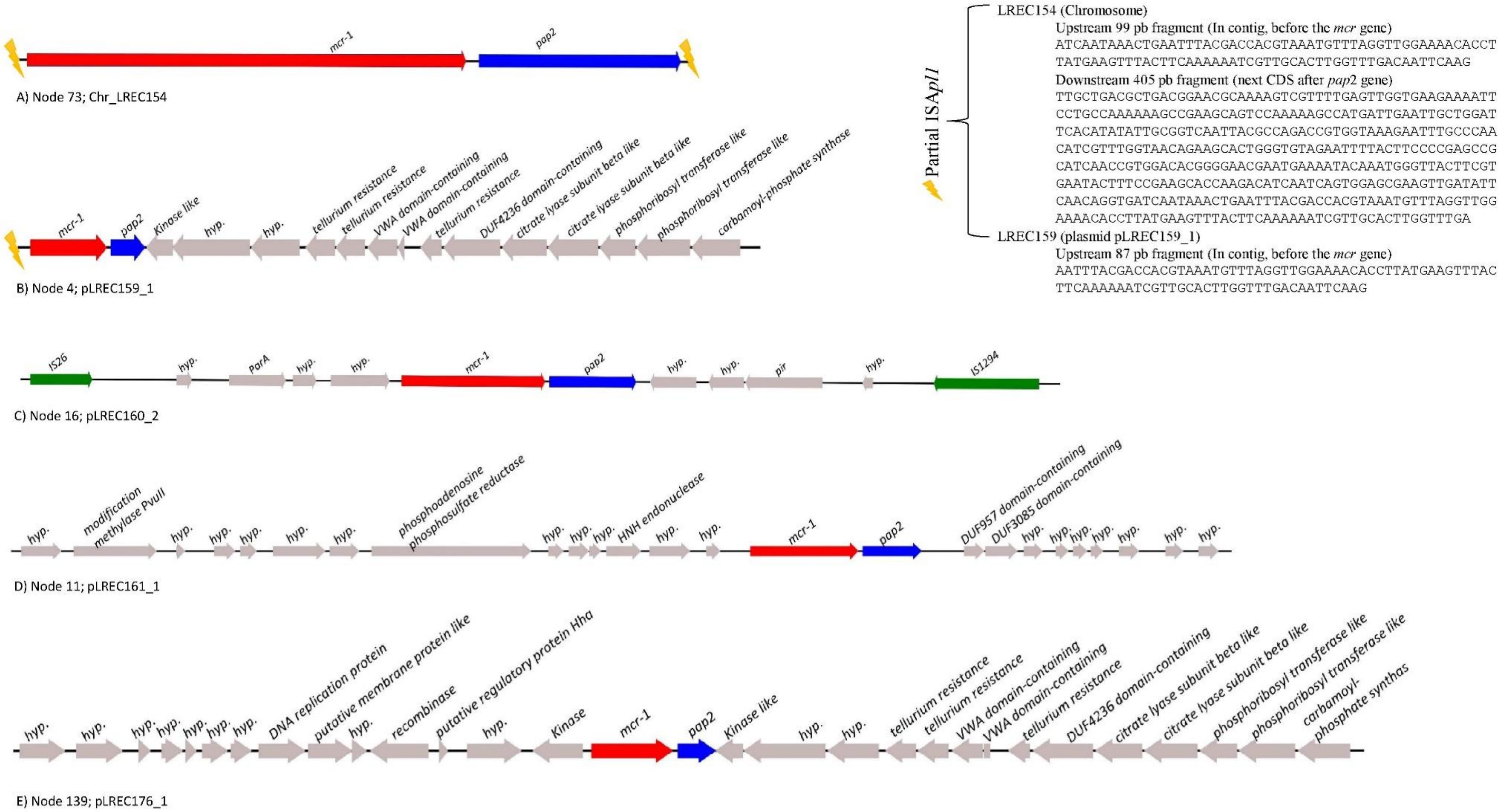

Supplementary Figure 17. Genetic environment of the *mcr*-1 gene found in the studied genomes. The *mcr*-1 gene is colored in red, the *pap2* gene in blue and the insertion elements in green. Hyp: hypothetical protein.

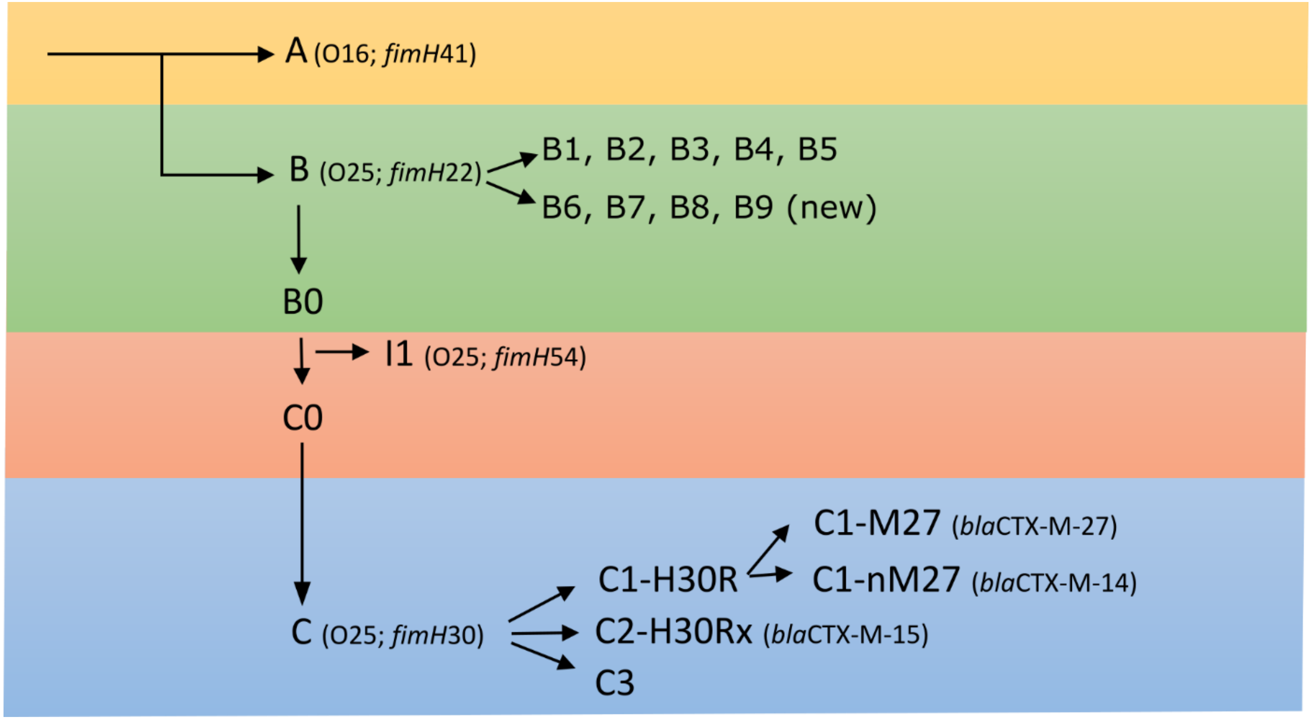

Supplementary Figure 18. Graphic representation of the ST131 lineage.

2 References

Ben Zakour, Nouri L, Areej S Alsheikh-Hussain, Melinda M Ashcroft, Nguyen Thi Khanh Nhu, Leah W Roberts, Mitchell Stanton-Cook, Mark A Schembri, and Scott A Beatson. 2016. “Sequential Acquisition of Virulence and Fluoroquinolone Resistance Has Shaped the Evolution of Escherichia Coli ST131.” *MBio* 7 (2): no pagination. https://doi.org/10.1128/mBio.00347-16.

Lanza, Val F., María de Toro, M. Pilar Garcillán-Barcia, Azucena Mora, Jorge Blanco, Teresa M. Coque, and Fernando de la Cruz. 2014. “Plasmid Flux in Escherichia Coli ST131 Sublineages, Analyzed by Plasmid Constellation Network (PLACNET), a New Method for Plasmid Reconstruction from Whole Genome Sequences.” Edited by Paul M. Richardson. *PLoS Genetics* 10 (12): e1004766. https://doi.org/10.1371/journal.pgen.1004766.

Matsumura, Yasufumi, Johann D. D. Pitout, Gisele Peirano, Rebekah DeVinney, Taro Noguchi, Masaki Yamamoto, Ryota Gomi, et al. 2017. “Rapid Identification of Different Escherichia Coli Sequence Type 131 Clades.” *Antimicrobial Agents and Chemotherapy* 61 (8): 1–9. https://doi.org/10.1128/AAC.00179-17.
